# Secretory proteins are delivered to the septin-organized penetration interface during root infection by *Verticillium dahliae*

**DOI:** 10.1371/journal.ppat.1006275

**Published:** 2017-03-10

**Authors:** Ting-Ting Zhou, Yun-Long Zhao, Hui-Shan Guo

**Affiliations:** 1 State Key Laboratory of Plant Genomics, Institute of Microbiology, Chinese Academy of Sciences, Beijing, China; 2 College of Life Sciences, University of the Chinese Academy of Sciences, Beijing, China; University of Exeter, UNITED KINGDOM

## Abstract

Successful infection of the host requires secretion of effector proteins to evade or suppress plant immunity. Secretion of effectors in root-infecting fungal pathogens, however, remains unexplored. We previously reported that *Verticillium dahliae*, a root-infecting phytopathogenic fungus, develops a penetration peg from a hyphopodium to infect cotton roots. In this study, we report that a septin ring, requiring VdSep5, partitions the hyphopodium and the invasive hypha and form the specialized fungus-host interface. The mutant strain, Vd*Δnoxb*, in which NADPH oxidase B (VdNoxB) is deleted, impaired formation of the septin ring at the hyphal neck, indicating that NADPH oxidases regulate septin ring organization. Using GFP tagging and live cell imaging, we observed that several signal peptide containing secreted proteins showed ring signal accumulation/secretion at the penetration interface surrounding the hyphal neck. Targeted mutation for VdSep5 reduced the delivery rate of secretory proteins to the penetration interface. Blocking the secretory pathway by disrupting the vesicular trafficking factors, *VdSec22* and *VdSyn8*, or the exocyst subunit, *VdExo70*, also arrested delivery of the secreted proteins inside the hyphopodium. Reduced virulence was observed when cotton roots were infected with Vd*Δsep5*, Vd*Δsec22*, Vd*Δsyn8* and Vd*Δexo70* mutants compared to infection with the isogenic wild-type V592. Taken together, our data demonstrate that the hyphal neck is an important site for protein secretion during plant root infection, and that the multiple secretory routes are involved in the secretion.

## Author summary

Pathogens secrete effector proteins as molecular weapons to evade or suppress plant immunity. However, the mechanism(s) by which root-infecting fungal pathogens secrete secretory effector proteins remains unexplored. We previously reported that *Verticillium dahliae*, a root-infecting phytopathogenic fungus, forms a specialized infection structure known as a hyphopodium that develops a penetration peg to pierce plant roots. In this study, we observed that after penetration, the penetration peg-developed hyphal neck, partitioning the hyphopodium and invasive hypha, came into close contact with the host, forming the fungus-host penetration interface. NADPH oxidase B (VdNoxB) regulated the cytoskeletal organization of the septin ring at the hyphal neck. Importantly, the penetration interface was a preferential site for secretion of signal peptide-containing proteins. Septin plays an important role in the efficient delivery of secretory proteins to the penetration interface. Moreover, the conventional fungal ER-to-Golgi secretion pathway, endosome-mediated transport and the exocyst complex are involved in the delivery of secretory proteins to the penetration interface. Together, our data demonstrate that the *V*. *dahliae* infection structure functions as a key signaling hub during plant infection and is the apparatus that not only breaches host cells but also provides a unique interface for the secretion of fungal effectors.

## Introduction

Pathogens secrete effector proteins as molecular weapons to evade or suppress plant immunity. Most effectors are small secreted proteins [1,2,3], and in many cases, the expression of these genes is induced by infection, helping the microbe to successfully colonize on the surface or inside of the host [1]. Studies of the secretion system have revealed diverse manners for pathogen effector translocation into their host. Fungi secrete different effectors at different infection stages from stage-specific compartments at the host-pathogen interface [4]. Before penetrating host cells, some effector proteins are focally secreted from appressorial penetration pores and may function to suppress early plant defense responses, as in case of several *Colletotrichum* species [4,5]. After invasive hypha developed, effectors in *Colletotrichum orbiculare* accumulate at the pathogen-plant biotrophic interface, a ring-like region around the neck of the biotrophic primary hypha [5]. Effectors of some plant fungal pathogens are putatively translocated into the host cell, where they interact with cytoplasmic or nuclear R proteins [6]. For example, *Magnaporthe oryzae* has a highly localized structure to accumulate cytoplasmic effectors secreted by invasive hyphae, known as the biotrophic interfacial complex (BIC), which forms at the tip of the initially filamentous hypha in the host cell [7,8].

Secretion of effectors to the host is also important for soil-borne fungal and oomycete pathogens, such as *Verticillium dahliae* and *Phytophthora sojae*, for successful infection [9,10]. Transit of many oomycete or fungal effectors to host cell depends only on the RXLR motif or other host-entry motifs of the effectors and host molecules, but not pathogen-encoded machinery [11,12]. However, however, the mechanism(s) by which root-infecting fungal pathogens secrete secretory protein remains unknown. *V*. *dahliae* causes vascular wilt disease [13,14] and infects more than 200 host species worldwide, including many economically important crops, such as lettuce, cotton and tomato [15]. *V*. *dahliae* contains more than 100 small cysteine-rich potentially secreted proteins [3]. So far, only two effectors, Ave1 and Vdlsc1, have been functionally studied. Ave1 contributes to fungal virulence in the absence of its corresponding R protein (Ve1) [9]; it is a small secreted protein containing 134 amino acids (aa) with four cysteines [9], however, its secretion process has not been studied. Vdlsc1 suppresses salicylate-mediated innate immunity *in planta* [10]. Vdlsc1 is an unconventionally secreted protein as it lacks an N-terminal signal peptide that can direct the protein to the conventional secretory pathway [10]. Therefore, whether the soil-borne *V*. *dahliae*, a root-infecting phytopathogenic fungus, have a highly localized structure to secret secretory effector proteins remains unexplored.

We recently identified and provided the molecular features of the infectious structure, the hyphopodium, in *V*. *dahliae* [16]. We demonstrate that *V*. *dahliae* NADPH oxidase B (VdNoxB) is required for local reactive oxygen species (ROS) production during infection, and ROS-Ca^2+^ signaling in the hyphopodium plays key roles in regulating polarized penetration peg formation and pathogenicity in *V*. *dahliae* [16]. In *M*. *oryzae*, the Nox2 (NoxB)-NoxR complex spatially organizes a heteroligomeric septin ring at the appressorium pore [17,18]. Septins, small morphogenetic guanosine triphosphatases (GTPases), are thought to reorient and reorganize the cytoskeleton to determine cell shape [19]. Septin ring scaffolds a toroidal F-actin ring and recruits and organizes the exocyst to the appressorium pore where the penetration peg emerges [17,18,20].

In this study, to explore whether the secretion of effectors in *V*. *dahliae* could be associated with the penetration peg, we first verified that VdNoxB was required for the cytoskeletal organization of a septin ring at the penetration peg and its derived hyphal neck in *V*. *dahliae*. We observed that the septin-ring-organized hyphal neck acts as a functional fungus-host penetration interface for the delivery and secretion of signal peptide-containing secretory proteins. Using gene functional analyses, we further showed that VdSep5, the conventional fungal ER-to-Golgi secretion pathway, the endosome-mediated transport, and the exocyst complex are involved in the delivery of secretory proteins to the penetration interface.

## Results

### VdNoxB-dependent penetration peg and its derived hyphal neck are repeatedly developed during *V*. *dahliae* infection of plant roots

**Fig 1:**
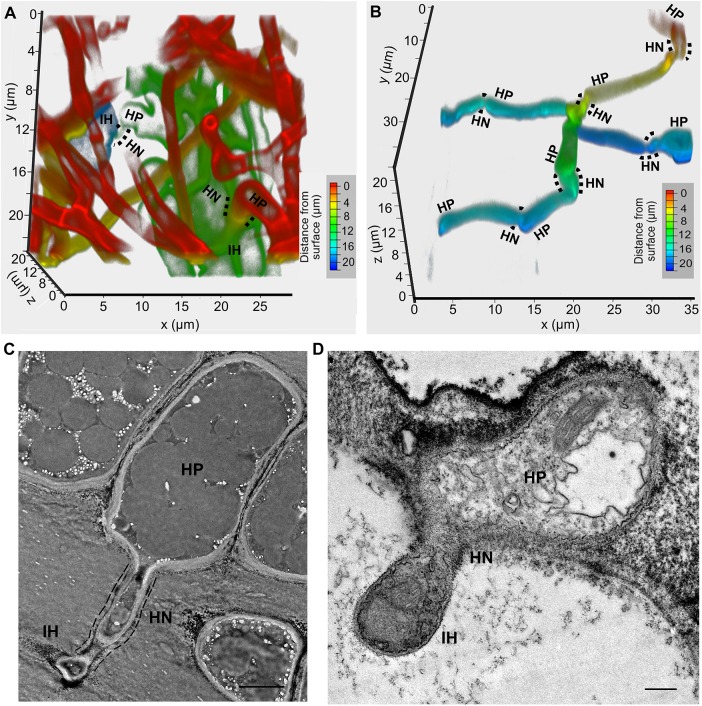
Penetration peg-derived hyphal neck partitioning the hyphopodium and invasive hypha. (A, B) Confocal laser scanning microscopy (CLSM) images of the development and penetration of hyphopodium of *V*. *dahliae* V592 on cellophane (A) and *Arabidopsis thaliana* root (B). The fungal cell wall was stained with FITC-WGA. Images were obtained at 7 dpi on cellophane and 1 dpi on roots. (C, D) Transmission electron microscopy analysis of *V*. *dahliae* invasion of cellophane (C) and cotton root (D). The dashed lines represent the penetration interface of the penetration peg-derived hyphal neck on cellophane. HP, hyphopodium; HN, hyphal neck; IH, invasive hypha. Bar = 2.5 μm in (C) and Bar = 0.5 μm in (D).

*Verticillium dahliae* infection requires the development of an infectious structure, hyphopodium, in which the NADPH oxidase catalytic subunit VdNoxB is specifically expressed to regulate formation of the penetration peg to pierce the cell wall [16]. To further understand the infection process, we used FITC-WGA (FITC-conjugated wheat germ agglutinin) to label the fungal hyphae of wild-type *V*. *dahliae* strain V592 to assess cellophane penetration and root infection. We observed repeated development of hyphopodium for penetration inside the cellophane and roots (Fig 1A and 1B, S1 and S2 Movies). Transmission electron microscopy (TEM) images showed that after penetration, the invasive hypha grew, and the penetration peg acted as a hyphal neck that partitioned the hyphopodium and invasive hypha and came into close contact with the cellophane (Fig 1C) or the host (Fig 1D), forming the fungus-host penetration interface. WGA staining showed various uneven hyphal neck lengths after piercing the cellophane or root cell wall (Fig 1A and 1B). The length of the hyphal neck reflects the length of the penetration peg and might be dependent on the piercing sites of different cells, such as the root epidermis or cortical cells, and the piercing angle. Repeated development of hyphopodium inside the cellophane was observed using V592 expressing GFP-tagged VdNoxB under the native promoter (S2A Fig) [16]. Inside the cellophane, the GFP signal was observed in flattened irregular hyphopodia and at the tips of the penetration pegs (S2B Fig). The VdΔ*noxb* mutant, in which *VdNoxB* was deleted, was incapable of producing a penetration peg (S2C Fig). Together, our results demonstrate that *V*. *dahliae* infection requires VdNoxB-dependent, repeated development of the hyphopodium and penetration peg for each cell wall penetration, and the penetration peg-derived hyphal neck connects the hyphopodium to the invasive hypha and marks a site of close fungus-host penetration interface contact.

### Organization of the cytoskeletal septin ring at the penetration peg and its derived hyphal neck

**Fig 2:**
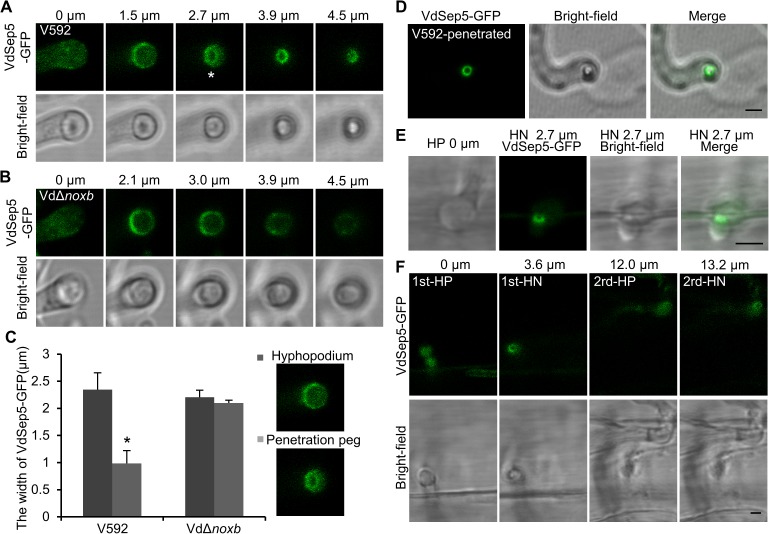
VdNoxB is required for the formation of a septin ring at the penetration peg and hyphal neck in *V*. *dahliae*. (A, B) Cellular localization of VdSep5-GFP in V592 and Vd*Δnoxb* during development of the penetration peg. Numbers indicate the distance from the center of the hyphopodium where the first column (0 μm) shows the beginning of a continuous z series. The star indicates the septin ring at the hyphopodium pore (2.7 μm). Bar = 2.5μm. (C) Quantitative analysis of the diameter reduction of the VdSep5-GFP ring. In V592, from the widest part at the base of the hyphopodium (the 1.5-μm plane) to the hyphopodium pore (the 2.7-μm plane); in VdΔ*noxb*, from the 2.1-μm plane to the 3.9-μm plane. The bar chart shows the average diameter of the fluorescence signal ring, and 20 hyphopodia were investigated in each assay with three replicates (*P<0.05; t-test). (D, E) VdSep5-GFP localized at the hyphal neck partitioning the hyphopodium and the invasion hypha on cellophane (D) or *A*. *Thaliana* roots (E). Bar = 2.5 μm. (F) The VdSep5-GFP ring localized at two individual penetration sites during root epidermis and cortical cell wall penetration. Bar = 2.5 μm.

To explore the specific features of the penetration peg and its derived hyphal neck, we first examined whether the cytoskeleton protein septin plays a role in determining penetration peg morphogenesis in *V*. *dahliae*. The *V*. *dahliae* homolog of Septin5 was identified (S1B Fig) and named VdSep5. VdSep5-GFP was expressed in the V592 and VdΔ*noxb* mutant. In the wild-type V592 hyphopodium during penetration peg induction, confocal laser scanning microscopy (CLSM) observation revealed a continuous funnel-shaped VdSep5-GFP fluorescent signal from the base of the hyphopodium, outlining the curved contact area between the hyphopodium and cellophane membrane (Fig 2A, plane 1.5 μm), to the central protruded zone (refer to the hyphopodium pore) where the penetration peg was initially developed and spread throughout to its tip (Fig 2A, from plane 2.7 to 4.5 μm). In contrast, in the VdΔ*noxb* hyphopodium, the VdSep5-GFP signal outlined the curved contact area between the hyphopodium and the cellophane membrane but without the central septin ring at the base of the hyphopodium (Fig 2B). In wild-type V592, compared with the widest part of the VdSep5-GFP signal area (Fig 2A, plane 1.5 μm), the diameter of the VdSep5-GFP ring at the hyphopodium pore (Fig 2A, plane 2.7 μm) was reduced approximately 58%, while there was no clear reduction of the diameter of the VdSep5-GFP signal area in the VdΔ*noxb* mutant, which is deficient in penetration peg formation (Fig 2C). After cellophane piercing and invasive hyphal growth, we observed the compact septin ring signal retained in the hyphal neck (Fig 2D). These results demonstrate that VdNoxB is required for VdSep5 organization of the cytoskeleton to determine the morphogenesis of the penetration peg and its derived hyphal neck.

After infecting *Arabidopsis* root with wild-type *V*. *dahliae* V592, a VdSep5-GFP ring was also observed at the hyphal neck partitioning the hyphopodium and invasive hyphae (Fig 2E). Two VdSep5-GFP rings were observed in the first and second hyphal necks in two CLSM planes within the same scanning view (Fig 2F, 3.6 μm for the first penetration and 13.2 μm for the second penetration), verifying the requirement of multiple penetrations for each new cell wall to reach the vascular bundle. Together, our results clearly demonstrate that septin-ring organization accompanies *V*. *dahliae* penetration of either cellophane or plant roots, and VdNoxB plays a role in the organization of the septin ring at the penetration peg and its derived hyphal neck. Remarkably, once established, the VdSep5-GFP ring was retained at and framed the hyphal neck, forming the fungus-host penetration interface.

Consistent with previous findings that septin scaffolds a toroidal F-actin ring at the appressorium pore in *M*. *oryzae* [17,18], we also observed that F-actin was organized as a ring structure at the hyphal neck in either cellophane or root by live-cell imaging of V592 expressing LifeAct-GFP (S3 Fig).

### Secretory proteins are preferentially localized at penetration interfaces

**Fig 3:**
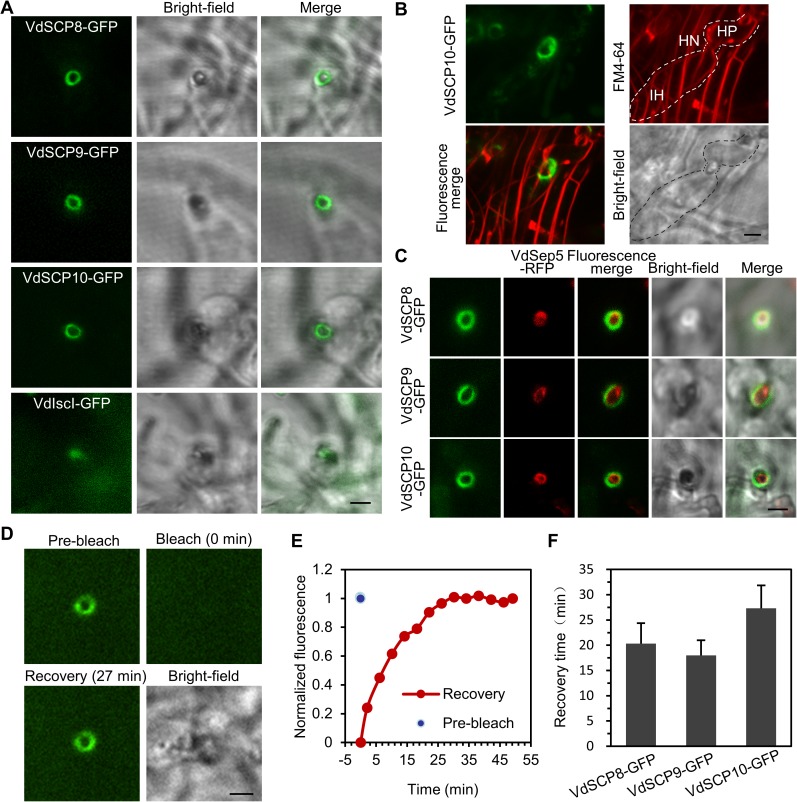
Accumulation of secretory proteins surrounding the hyphal neck on cellophane. (A) VdSCP8-GFP, VdSCP9-GFP and VdSCP10-GFP but not VdIscI-GFP, showed ring signal accumulation at the hyphal necks. Images were obtained at 8 days after the fungal strains were incubated on M0 medium overlaid with cellophane, in each invasive hyphae were observed. Bar = 2.5 μm. (B) The VdSCP10-GFP ring signal surrounds hyphal neck. The hyphal plasma membrane was stained with FM4-64 (red). VdSCP10-GFP, FM4-64, and fluorescence merge images are z-series stacks. HP, HN and IH are marked in the bright-field picture. Bar = 2.5 μm. (C) VdSCP8-GFP, VdSCP9-GFP and VdSCP10-GFP rings localized outside of the VdSep5-RFP ring. Bar = 2.5 μm. (D) FRAP detection of the dynamic accumulation/delivery of VdSCP10-GFP at the hyphal neck on cellophane. Fluorescence at the penetration interface (Pre-bleach) was photobleached (Bleach) and allowed to recover 100% for 27 min (Recovery). Bar = 2.5 μm. (E) Plot of normalized penetration interface fluorescence intensity recovery over time for VdSCP10-GFP. (F) The average recovery time of VdSCP8-GFP, VdSCP9-GFP and VdSCP10-GFP after bleaching on cellophane. Three FRAP tests were performed for each sample.

Next, we investigated whether the septin-ring-organized hyphal neck, in addition to its piercing role, could act as a functional fungus-host penetration interface for the delivery of secretory proteins. The arsenal of potentially secreted proteins in plant pathogens includes key pathogenicity molecules that are generally referred to as effectors (small cysteine-rich proteins, <400 amino acids (aa) and ≥4 cysteine residues) [3]. Because the well-known Ave1 secreted effector has not been identified in the cotton isolate V592, we selected three small cysteine-rich proteins (SCP), VDAG_08085 (194 aa, 6 Cys, named VdSCP8), VDAG_00902 (375 aa, 16 Cys, VdSCP9) and VDAG_05717 (205aa, 4 Cys, VdSCP10), for analysis in this study. Each of these SCPs has an N-terminal signal peptide predicted by the SignalP 4.1 server (S4A Fig) [21]. Among them, VdSCP9 is a LysM domain-containing protein. The LysM effector family contains relatively conserved secretory proteins that are known to play significant roles in the pathogen-host interaction [3,22,23,24]. VdSCP8 was identified by liquid chromatography-mass spectrometry (LC-MS) of the V592 culture filtrate, and VdSCP10 was one of the potential pathogenicity genes in our previous screening of the T-DNA insertional mutant library [25] and confirmed by the targeted gene replacement mutant of VdSCP10 (S5 Fig). Transcript levels of these SCP genes were first examined using quantitative RT-PCR (qRT-PCR). The expression levels of *VdSCP9* and *VdSCP10*, but not *VdSCP8*, were significantly up-regulated at 4 days post-inoculation (dpi) of V592 on cellophane and at 2 dpi on cotton roots (S4B Fig). To observe the localization of these SCPs, VdSCP8-GFP, VdSCP9-GFP and VdSCP10-GFP were expressed under the native promoter. Only VdSCP8-GFP fluorescence was detectable as a ring signal at the penetration zone on cellophane (S4C Fig). Neither VdSCP9-GFP nor VdSCP10-GFP fluorescence was observed on cellophane. Therefore, these GFP fusion proteins were constructed under the oliC promoter. The *V*. *dahliae* small effector VdIsc1 (190 aa, 1 Cys), which lacks a signal peptide and exhibits characteristics that lead to unconventional secretion [10], was also fused to GFP as a control. After *V*. *dahliae* invasion into cellophane, VdSCP8-GFP, VdSCP9-GFP and VdSCP10-GFP, but not the control VdIsc1-GFP, showed ring signals surrounding the penetration zones (Fig 3A). From a picture of the penetration at an incline, the VdSCP10-GFP ring signal was clearly observed surrounding the hyphal neck linking the hyphopodium to the invasive hypha (Fig 3B).We also fused the signal peptides of SCPs to GFP and found that SP_VdSCP8_-GFP, SP_VdSCP9_-GFP and SP_VdSCP10_-GFP also showed ring signals outside the plasma membrane of the penetration zones (S4D Fig). To detect whether the SCP-GFP signal rings overlapping with the septin ring, VdSep5-RFP was co-expressed with VdSCP8-GFP in V592, and the results showed that the VdSep5-RFP ring was inside the VdSCP8-GFP ring (Fig 3C). Similar results were obtained for VdSCP9-GFP and VdSCP10-GFP, demonstrating that signal peptide-containing SCPs accumulate and/or are delivered to the hyphal neck for secretion. Together, these data suggest that the hyphal neck made up a fungus-host penetration interface for the delivery and/or exportation of secretory proteins.

**Fig 4:**
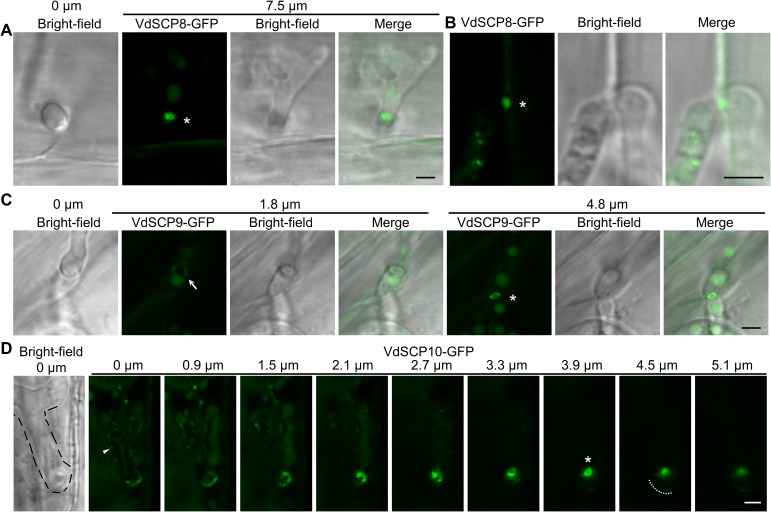
Accumulation of secretory proteins at the hyphal neck during root colonization. (A) VdSCP8-GFP localized at the hyphal neck (at 7.5 μm, distance from the center of the hyphopodium where the first image was obtained at 0 μm), joining a lightly melanized hyphopodium and an invasive hypha. (B) VdSCP8-GFP ring signal accumulated at the cell junction. (C) VdSCP9-GFP shows a weak ring signal at the first hyphal neck (at 1.8 μm, arrow) and a strong ring signal at the second hyphal neck (at 4.8 μm) in two individual penetrations. (D) Z-series projection showing that VdSCP10-GFP preferentially localized at the hyphal neck on roots. Arrowhead indicates VdSCP10-GFP signal dots inside hyphopodium. Black dashed line outlining the hyphopodium in the first picture and white dashed line marking the beginning of an invasive hypha. Fluorescence micrographs of (A,B,C) were merged from 2–3 continuous z series images. Asterisks indicate the ring signals. Bar = 2.5 μm.

Next, we inoculated strains of V592 expressing the GFP-tagged SCPs on *Arabidopsis* roots. VdSCP8-GFP accumulation was first observed at approximately 2 dpi. A strong VdSCP8-GFP signal ring was observed at the hyphal neck, which partitioned the hyphopodium and the invasive hypha (Fig 4A and 4B). Some weak signals were also observed inside the invasive hyphae (Fig 4A and 4B). VdSCP9-GFP accumulation was first observed at 1 dpi. One weak and one strong VdSCP9-GFP signal ringwas observed, respectively, at the first and second hyphal neck in two CLSM planes in the same scanning view (Fig 4C at planes of 1.8 μm and 4.8 μm). This observation is consistent with the requirement of the repeated development of the hyphopodium for each cell wall penetration during the colonization of V592 from the root surface to the vascular bundle (Fig 1B). The VdSCP10-GFP ring signal was observed at approximately 6 dpi. In the upper plane of the hyphopodium (0 μm), a weak VdSCP10-GFP speckle signal was observed at the periphery of the hyphal cell and on two sides of a septa (Fig 4D). A stronger signal was observed at the apex of the hyphopodium (Fig 4D). The clear and strongest VdSCP10-GFP ring signal was observed at and throughout the hyphal neck using a series of continuous scanning planes (Fig 4D). Taken together, our data demonstrate that successful invasion of plant roots and cellophane by *V*. *dahliae* has the common phenomenon of signal peptide-containing secretory protein recruitment at the hyphal neck for effective secretion through this fungus-host penetration interface. The detectable ring signals for each secretory protein at different time points suggest that their synthesis and/or rate of delivery were different, revealing a complex process for successful infection in plant roots by *V*. *dahliae*.

We then examined whether the directional ring-shaped accumulation of the small secretory proteins was derived from dynamic secretion towards the penetration interface. Fluorescence recovery after photobleaching (FRAP) was performed with the V592 strain expressing VdSCP10-GFP on cellophane at 6 dpi. We photobleached VdSCP10-GFP fluorescence at the penetration site and then monitored the fluorescence recovery over time. After near complete elimination, fluorescence recovered within 27 min (Fig 3D and 3E). The fluorescence recovery time for VdSCP8-GFP and VdSCP9-GFP was 22 min and 18 min, respectively (Fig 3F). These data suggest that secretory proteins were continuously synthesized and/or delivered to the penetration interface.

### VdSep5 plays a role in the delivery of protein secretion toward the penetration interface

To detect whether septin also plays a role in the delivery of secretory proteins to penetration interfaces, the targeted gene knockout mutants VdΔ*sep5* and VdΔ*sep3* were generated in wild-type V592 (S6A and S6B Fig). The VdSep3 homologous sequence was searched from V592 based on a BLASTP search using MoSep3 and the VdLs.17 database (S1A Fig). The *VdSep3* knockout mutant strain exhibited a reduced hyphal growth rate on PDA medium compared with V592 (S6D Fig), and developed an abnormal hyphopodium on cellophane without smooth swelling at the end of branching hypha (S6F Fig) that was incapable of forming a penetration peg to pierce the cellophane (S6E Fig). This result demonstrates that VdSep3 plays roles in hyphal growth and hyphopodium development. In contrast, the *VdSep5* knockout mutant strain exhibited a normal growth rate on PDA medium (S6D Fig) but developed fewer hyphopodia on cellophane (S6F and S6G Fig) and displayed greatly delayed penetration of the cellophane compared with V592 (S6E Fig), demonstrating that VdSep5 plays a role in hyphopodium development. Consistently, both the VdΔ*sep3* and the VdΔ*sep5* mutant showed reduced virulence on cotton plants (S6H and S6I Fig). The reintroduction of P*sep3*:VdSep3:T*trpc* and P*tef*:VdSep5-GFP:T*trpc* restored the hyphal morphologies and cellophane penetration abilities, as well as the pathogenicity (S6D, S6E, S6H and S6I Fig), confirming the targeted gene deletion. Our results suggest that VdSep5 plays an important role in the initiation of hyphopodium formation, whereas, VdSep3 is more important for development of the hyphopodium.

**Fig 5:**
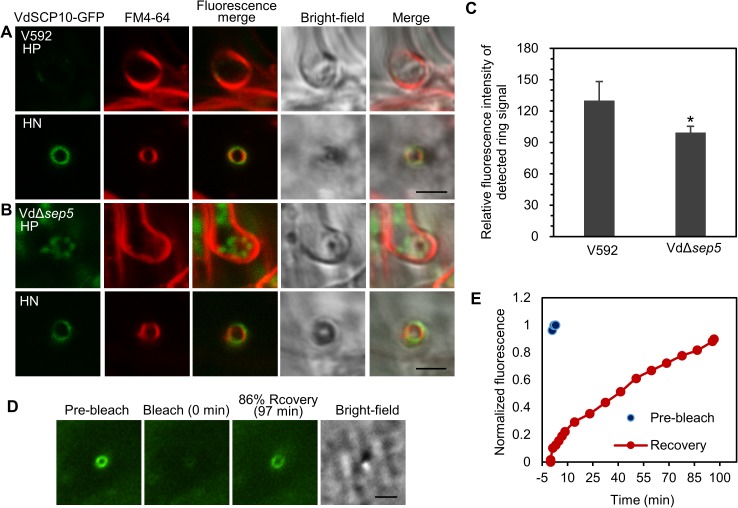
Deletion of *VdSep5* decreased secretory protein delivery to the hyphal neck. (A, B) VdSCP10-GFP signal ring surrounding the hyphal neck in wild-type V592; mutant strain Vd*Δsep5* retained most of the VdSCP10-GFP inside the hyphopodium and reduced the VdSCP10-GFP signal at the hyphal neck. The plasma membrane of HP and HN was stained with FM4-64 (red). Bar = 2.5 μm. (C) Quantitative analysis of the effect of VdSep5 on VdSCP10-GFP delivery to the penetration interface. More than 30 hyphal necks with a visible signal were investigated for each strain to determine the intensity of the ring signal at the hyphal neck. Two Vd*Δsep5* mutant strains obtained from individual VdSCP10-GFP-expressing V592 (S6B Fig) were used for the observation. The mean and SD were calculated from two VdSCP10-GFP-expressing V592 and the corresponding Vd*Δsep5* strains with two biological repeats (*P<0.05; t-test). (D, E) FRAP assay for the dynamic accumulation/delivery of VdSCP10-GFP at the hyphal neck in Vd*Δsep5*. Three FRAP tests were performed.

To observe the localization of secretory protein in the VdΔ*sep5* mutant, the targeted gene knockout mutant was generated in VdSCP10-GFP-expressing V592 strain. VdSCP10-GFP secretion in the *VdSep5* deletion mutant was assessed. In contrast to the remarkable VdSCP10-GFP signal ring surrounding the hyphal neck in the wild-type V592 (Fig 5A), the VdSCP10-GFP signal was observed in both the hyphopodium and hyphal neck in the VdΔ*sep5* mutant, in which either the hyphopodium or the hyphal neck was stained with FM4-64 (Fig 5A and 5B). The VdSCP10-GFP signal at the hyphal neck in the VdΔ*sep5* mutant was clearly reduced compared with that in the wild-type V592 background (Fig 5B). The average signal intensity of VdSCP10-GFP in the hyphal neck of VdΔ*sep5* was approximately 78% of that in V592 (Fig 5C). The FRAP assay showed 86% recovery of VdSCP10-GFP fluorescence at the penetration interface within 97 min (Fig 5D and 5E), which was significantly longer than the recovery time of 27 min for the wild-type V592. Three FRAP tests on cellophane showed a similar delayed in secretion. These results demonstrate that VdSep5 plays a role in mediating the delivery of secretory proteins to the penetration interface, in addition to its functions in hyphopodium development and cortical structure organization of the penetration peg and hyphal neck.

### The vesicular trafficking factor SNAREs VdSec22 and VdSyn8 are involved in protein secretion toward penetration interfaces

We next investigated the role of vesicular traffic in the delivery of secretory proteins to penetration interfaces. SNAREs function as key elements in membrane fusion [26,27,28]. The R-SNARE Sec22 is important for modulating transport between the ER and the Golgi apparatus [29]. The Qc-SNARE Syn8 in *S*. *cerevisiae* and *M*. *oryzae* (MoSyn8) localizes at endosomes and/or late endosome/prevacuolar compartments (PVCs) [26,28]. To identify functional proteins in the secretion of *V*. *dahliae*, homologous sequences were searched in V592 based on a BLASTP search using MoSec22 and MoSyn8 and the database for VdLs.17, designated VdSec22 and VdSyn8, respectively (S1C and S1D Fig). Targeted gene knockout mutants VdΔ*sec22* and VdΔ*syn8* were generated (S7A and S7B Fig). Both mutants exhibited growth defects with a reduced vegetative hyphal growth rate; VdΔ*syn8* also showed reduced melanin production (S7D Fig). The reintroduction of VdSec22 and VdSyn8 under the control of each native promoter recovered the growth ability and hyphal morphologies (S7C and S7D Fig), confirming the targeted gene deletion of *VdSec22* and *VdSyn8*.

**Fig 6:**
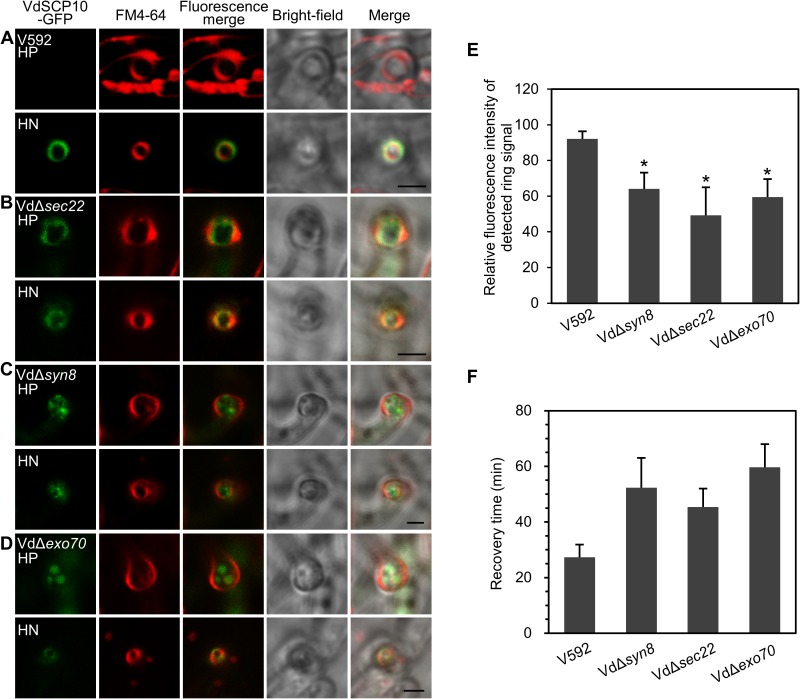
Deletion of *VdSec22*, *VdSyn8* or *VdExo70* decreased secretory protein delivery to the hyphal necks. (A) VdSCP10-GFP signal ring surrounding the hyphal neck in wild-type V592. (B-D) The mutant strains Vd*Δsec22* (B), Vd*Δsyn8* (C) and Vd*Δexo70* (D) retained most of the VdSCP10-GFP inside the hyphopodium and reduced the VdSCP10-GFP signal in the hyphal neck. The plasma membrane of HP and HN was stained with FM4-64 (red). (E) Quantitative analysis of the effect of VdSec22, VdSyn8, and VdExo70 on secretory protein delivery to penetration interfaces. More than 30 hyphal necks with a visible “ring” signal were investigated for each VdSCP10-GFP-expressing mutant strain to determine the intensity of the ring signal at the HN. The mean and SD for (E) were calculated from three independent fungal transformants for each mutant (*P<0.05; t-test). Bar = 2.5 μm. (F) FRAP assay for the dynamic accumulation/delivery of VdSCP10-GFP at the hyphal neck in Vd*Δsec22*, Vd*Δsyn8* and Vd*Δexo70* on cellophane. Three FRAP tests were performed for each mutant strain.

VdSCP10-GFP secretion in the VdΔ*sec22* and VdΔ*syn8* mutants was assessed. VdSCP10-GFP expressed under the oliC promoter was transformed into VdΔ*sec22* and VdΔ*syn8* mutants. The single copy insertion strains determined by Southern blot were used for further analysis (S8 Fig). In contrast to the remarkable VdSCP10-GFP signal ring in wild-type V592 (Fig 6A), the VdSCP10-GFP signal was observed in both the hyphopodia and hyphal necks in both deletion mutant strains (Fig 6B and 6C). The VdSCP10-GFP signal in the hyphal neck in both mutants was also clearly reduced, and most of the VdSCP10-GFP signal rings were overlapping with or inside the plasma membrane compared with that in the wild-type V592 background (Fig 6B and 6C). The average signal intensity of VdSCP10-GFP in the hyphal neck of VdΔ*sec22* and VdΔSyn8 was approximately 54% and 70%, respectively, of that in V592 (Fig 6E), suggesting that ER-Golgi transport is a predominant route of transport of SCPs. The FRAP assay was also performed with VdSCP10-GFP-expressing VdΔ*sec22* and VdΔ*syn8* mutants. Fluorescence was recovered after approximately 52 min and 45 min in VdΔ*sec22* and VdΔ*syn8* mutants, respectively (Fig 6F), which was much longer than the recovery time of 27 min observed for wild-type V592. Our data demonstrate that VdSec22 and VdSyn8 play roles in mediating the delivery of secretory proteins to the penetration interface.

To further determine whether the VdSCP10-GFP signal in the hyphopodium and hyphal neck was due to decreased transport from the ER to the Golgi apparatus in the mutant strains, a VdSCP10-GFP-expressing VdΔ*sec22* mutant on cellophane was stained with ER-Tracker Blue-White DPX. The VdSCP10-GFP signal was observed to overlap with the ER in the hyphopodium (S9 Fig), suggesting that the deletion of *VdSec22* resulted in retention of VdSCP10-GFP in the ER.

Taken together, our data demonstrate that the transport route between the hyphal ER and Golgi apparatus and endosome-mediated transport are involved in protein secretion toward penetration interfaces.

### Efficient secretion of secretory proteins at the penetration interface requires the exocyst complex

The exocyst was discovered as a tethering complex that mediates the initial encounter of arriving exocytic vesicles with the plasma membrane [30]. The exocyst complex is an evolutionarily conserved doctameric protein complex comprising Sec3, Sec5, Sec6, Sec8, Sec10, Sec15, Exo70, and Exo84 [31,32]. To test the role of the exocyst in the accumulation of small secretory proteins at the penetration interface in *V*. *dahliae*, two predicted exocyst components, VdSec8 and VdExo70, were identified (S1E and S1F Fig). VdSec8-GFP and VdExo70-GFP were expressed under either their native promoter or the oliC promoter and introduced into V592. Similar localization profiles were observed for both GFP-tagged proteins under either the native or the oliC promoter; however, the GFP signal derived from the native promoter was weak, and thus the fluorescence signals derived from the oliC promoter were photographed. Both VdSec8-GFP and VdExo70-GFP were observed as a crescent structure at the growing tip of vegetative hyphae (S10A Fig). VdSec8-GFP and VdExo70-GFP were organized as a ring at the base of the hyphopodium that was observed before penetration peg formation on either cellophane or *Arabidopsis* root (S10B and S10C Fig). After the development of invasive hyphae, VdSec8-GFP was organized at the hyphal neck on either cellophane or root (S10D Fig). Together, our data demonstrate that the exocyst is active at the base of the hyphopodium and the hyphal neck.

To characterize the localization relationship between the exocyst complex and VdSep5, VdSep5-RFP was transformed into the V592-expressing P*olic*:VdSec8-GFP:T*trpc* strain. Red septin rings were observed in all 20 observed hyphal necks, and VdSec8-GFP signal rings were observed in 14 of the detected septin rings. The corresponding linescan confirmed the co-localization of VdSec8-GFP and VdSep5-RFP (S11 Fig).

To further study the role of exocyst subunits on secretory protein accumulation at the penetration interface, we tried to knockout *VdExo70* and *VdSec8* in V592. VdΔ*exo70* mutants carrying the *VdExo70* deletion were obtained (S7B Fig), but the deletion of *VdSec8* was not successful, in agreement with a previous study in which *M*. *oryzae* exocyst-encoding gene knockouts generated only Δ*sec5* and Δ*exo70* mutants [20,33]. Thus, the failure to delete *VdSec8* was possibly due to the lethality of the absence of Sec8 in filamentous fungi including *V*. *dahliae* and *M*. *oryzae*. The VdΔ*exo70* mutant exhibited growth defects with a low growth rate on PDA medium (S7D Fig). The reintroduction of P*olic*:VdExo70-GFP:T*trpc* into the VdΔ*exo70* mutant recovered the growth ability and hyphal morphologies (S7C and S7D Fig), confirming the targeted disruption of *VdExo70*. VdSCP10-GFP was then transformed into the VdΔ*exo70* mutant and incubated on cellophane for hyphopodium induction. The single copy insertion strains determined by Southern blot were used for further analysis (S8 Fig). The VdSCP10-GFP signal was observed inside of the hyphopodium in the VdΔ*exo70* mutant (Fig 6D). Weak signals were observed in the hyphal neck, but most of them overlapped with the FM4-64-stained plasma membrane ring (Fig 6D). The average intensity of the green fluorescence ring of VdSCP10-GFP in the VdΔ*exo70* mutant was approximately 65% of that in V592 (Fig 6E). These results demonstrate that VdExo70 plays a role in secreting VdSCP10-GFP out of the hyphal neck. The FRAP assay on cellophane was also performed with VdSCP10-GFP in the VdΔ*exo70* mutant. The fluorescence recovered after 63 min (Fig 6F), which was significantly longer than the recovery time of 27 min determined for wild-type V592. Taken together, our results demonstrate that exocyst components also organize at the hyphal neck and take part in the delivery of secretory proteins to penetration interfaces.

### Proteins involved in the secretion pathway play critical roles in the pathogenicity of *V*. *dahliae*

**Fig 7:**
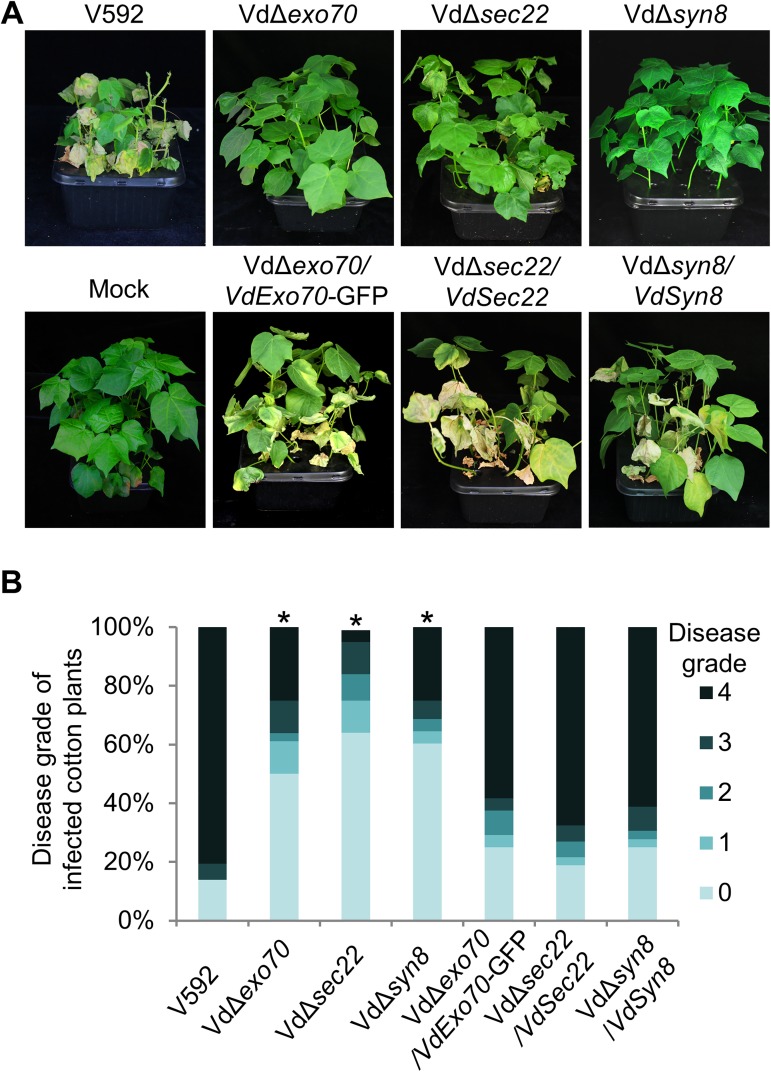
The exocyst subunit VdExo70 and SNAREs VdSec22 and VdSyn8 function in wilt virulence. (A, B) Disease symptoms (A) and disease grades (B) of cotton plants infected with wild-type V592, and Vd*Δexo70*, Vd*Δsec22* or Vd*Δsyn8* mutants and the complementary strains at 21 dpi. The grades were evaluated with three replicates of 36 plants for each inoculum (*P<0.05; t-test).

To explore the roles of proteins involved in the secretion pathway in the pathogenicity of *V*. *dahliae*, we inoculated VdΔ*exo70*, VdΔ*sec22* and VdΔ*syn8* mutants on cotton plants and found a significant (*P*< 0.05) reduction in the disease index for the three mutants (Fig 7A and 7B). The loss of virulence was restored when the VdΔ*exo70*, VdΔ*sec22* and VdΔ*syn8* mutants were complemented with P*olic*:VdExo70-GFP:T*trpc*, P*sec22*:VdSec22:T*trpc* or P*syn8*:VdSyn8:T*trpc*, respectively (Fig 7A and 7B). The significant loss of pathogenicity for the VdΔ*exo70*, VdΔ*sec22* and VdΔ*syn8* mutants was presumably consistent with their inefficient secretion of effector-related secretory proteins, which are required for successful fungal pathogen infection by evading or suppressing host plant immunity. Therefore, attributed to critical roles in the efficient secretion of secretory proteins at fungus-host penetration interfaces, VdExo70, VdSec22 and VdSyn8 play important roles in the pathogenicity of *V*. *dahliae*.

## Discussion

### Septin assembly at the penetration peg-derived hyphal neck framing an interface between *V*. *dahliae* hypha and the host

Penetration of the intact cuticles of the host is a very important step for successful infection by phytopathogens, either for leaf- or root-infecting fungal pathogens, such as *M*. *oryzae* and the anthracnose disease-causing *Colletotrichum* species or *V*. *dahliae* [4,5,16,17,34]. The foliar fungal pathogen *M*. *oryzae* forms conspicuous melanized appressoria with an average diameter of 8.0 μm when it inflates to full turgor and develops penetration pegs with an average diameter of 780-nm to breach the hydrophobic, waxy leaf cuticle [18,35]. In contrast, we found herein that the root-infecting fungus *V*. *dahliae* developed hyphopodia with an average diameter of 3.4 μm and formed a penetration peg with an average diameter of 1.3-μm, suggesting that less pressure is needed for *V*. *dahliae* to breach the root cuticle (Fig 1C and 1D).

We observed that the nature of the interface between *V*. *dahliae* hyphae and the host is the penetration peg-derived hyphal neck, in which a septin ring was organized. In *M*. *oryzae*, septins are found to provide the cortical rigidity and membrane curvature necessary for protrusion of the rigid penetration peg to breach the leaf surface [18]. Similarly, we found that the septin ring framed a recognizable cytoskeletal region of the hyphal neck in which F-actin was also organized as a ring structure, partitioning the hyphopodium and invasive hypha on both cellophane and roots. On cellophane, we also observed a funnel-shaped septin structure prior to invasive hyphal growth, suggesting that *V*. *dahliae* septins also function in the membrane curvature necessary for protrusion of the penetration peg at the base of the hyphopodium. In the *VdNoxB* knock out mutant, the VdSep5-GFP signal at the base of the hyphopodium suggests that septins provide membrane curvature, but the mutant strain failed to show protrusion of the penetration peg in the absence of VdNoxB. Previous studies in yeast and in fungal pathogen *Aspergillus fumigatus* suggest the importance of septin phosphorylation/dephosphorylation in controlling septin assembly [36,37]. In yeast, Rts1, a protein phosphatase 2A (PP2A) subunit, regulates septin dephosphorylation during telophase, and this dephosphorylation contributes to cytokinesis [36]. Dephosphorylation of the core septin, AspB, in a PP2A-dependent manner also impacts hyphal septation in *A*. *fumigatus* [37]. In animals, PP2A is a well-known tumor suppressor. ROS accumulation in cancer cells causes nitration and inactivation of PP2A, which interferes with the interaction of Bcl-2 with the PP2A catalytic core, leading to increased phosphorylation and antiapoptotic activity of Bcl-2 [38]. We recently reported that *V*. *dahliae* VdNoxB is required for local ROS production during infection and plays key roles in regulating polarized penetration peg formation [16]. Together with the regulated synthesis of ROS by *M*. *oryzae* Nox complexes directly control septin and F-actin dynamics [17], and the septin ring assembles in a kinase Chm1-dependent manner [18], we speculate that fungal Nox-dependent ROS might also play a role in inactivation of PP2A-like phosphatase, leading to increased Chm1-dependent septin phosphorylation, which is key for controlling septin assembly.

We speculate that septins also provide membrane curvature for polarity determination during penetration peg development on roots, although funnel-shaped septin signal was barely observed in the hyphopodium-penetration peg on the infected root, presumably due to a fast piercing process on the roots. The targeted gene deletions of VdSep5 or VdSep3 exhibited defects in hyphopodium and/or hyphal development, suggesting that core *V*. *dahliae* septins also act cooperatively to form heteroligomers during hyphal growth and infection. This result is consistent with previous observation in *M*. *oryzae* that septins formed rings at the neck of nascent appressoria and a wider range of structures in hyphae and during invasive growth, including bars, gauzes, collars and rings [18], in addition to an appressorium pore-located large septin ring [18]. Nevertheless, our data demonstrate the requirement for VdNoxB-dependent ROS in the regulation of cytoskeleton septin ring remodeling at the base of the hyphopodium, leading to rapid polarized growth of the penetration peg in *V*. *dahliae*. Each occurrence of penetration requires septin ring organization at the penetration peg and hyphal neck, supporting that successful colonization of extracellular hyphae to the vascular bundle requires repeated development of the hyphopodium and penetration peg, which repeatedly form penetration interfaces between *V*. *dahliae* hyphae and the host.

### The hyphal neck-associated penetration interface as the site of delivery of secretory proteins

Plant infection by pathogens involves the deployment of effector proteins that suppress plant immune responses and facilitate proliferation of the pathogen within plant tissues [30,35]. The delivery of effectors has been shown by extra-invasive hyphal membrane (EIHM) and BIC in the first-differentiated bulbous invasive hyphae in *M*. *grisea* [7,33]. In *C*. *higginsianum*, sequential delivery of host-induced effectors by the appressorium pore and intracellular hyphae has been observed [4]. In *C*. *orbiculare*, the accumulation of effectors occurred in the ring-like region around the neck linking the penetration peg to the biotrophic primary hyphae [5]. In this study, we found that during the penetration of cellophane or plant roots, the tested SCP-GFP and SP-GFP accumulated on the penetration interfaces, indicating a general role of the penetration interface as an active secretory protein delivery zone in *V*. *dahliae*. The FRAP assay revealed the dynamic accumulation of SCPs at the penetration interfaces. Secretion of the three SCPs into the hyphal neck is likely not dependent on the biological host. However, the cellophane membrane was used to mimic the hydrophobic niche for induction of appressoria in *M*. *grisea* [39] and hyphopodia in *V*. *dahliae* [16]. Together with the identification of VdSCP8 by LC-MS of the V592 culture filtrate without any treatment, and transcripts of *VdSCP9* and *VdSCP10*, but not *VdSCP8*, were induced upon incubation of *V*. *dahliae* on both cellophane and roots, we speculate that both VdSCP9 and VdSCP10 are probably *in planta*-expressed secretory proteins in *V*. *dahliae*. Moreover, the targeted gene deletion of *VdSCP10* caused a significant decrease in virulence toward cotton plants, suggesting that VdSCP10 may function as an effector to suppress plant immune responses. Although the LysM effector family contains relatively conserved secretory proteins that are known to play significant roles in the pathogen-host interaction [3,22,23], it has been recently reported that deletion of the VdSCP9 homologous core LysM protein, Vd4LysM, in *V*. *dahliae* strain JR2, did not compromise virulence during infection in *Arabidopsis*, tomato or *Nicotiana benthamiana* [24]. Whether VdSCP9 and VdSCP8 function as effectors to suppress plant immune responses or facilitate proliferation of *V*. *dahliae* within plant tissues requires further investigation. Remarkably, the ring signals of the tested SCPs were outside and around the hyphal neck and septin ring (Fig 3), and they were reduced in the hyphal neck in the VdΔ*sep5* mutant. These observations indicate that septins are not only required to organize the hyphal neck to form a fungus-host interface but also participate in the delivery and exportation of secretory proteins.

### Delivery of secretory proteins to the penetration interface via vesicular trafficking coupled with exocytosis

Phytopathogenic fungi express numerous small proteins that possess classical N-terminal signal peptides that direct them to the endoplasmic reticulum (ER) [3,4,40] The three signal peptide-containing SCPs, but not the unconventional secretion protein VdIscI, accumulated around the hyphal neck, suggesting that secretion into the penetration interface depends on ER processing. The retention of VdSCP10-GFP in the ER of the VdΔ*sec22* mutant demonstrates the importance of transport between the ER and the Golgi apparatus in secretory protein delivery to penetration interfaces in *V*. *dahliae*. In *M*. *oryzae* during the invasion of rice cells, ER-to-Golgi trafficking is involved in the secretion of apoplastic effectors by EIHM [33]. The Δ*sec22* mutants of *C*. *orbiculare* also show a decreased accumulation of effectors at biotrophic interfaces [5]. Similar to *M*. *oryzae* and *C*. *orbiculare* [5,41], the absence of Sec22 weakens the virulence of *V*. *dahliae* (Fig 7), suggesting that conventional ER-Golgi transport has a conserved function in the transport of some pathogen secretory proteins to interact with host molecules.

Endosomes participate in endocytosis and secretion during fungal infection in the host [28,42]. The long-distance retrograde motility of early endosomes is necessary to perceive plant cues and trigger the transcription of effector-coding genes during plant infection by the pathogenic fungus *Ustilago maydis*, which regulates effector production and secretion during host cell invasion [43]. Syn8 in *M*. *oryzae* is involved in the secretion of BIC-localized AVR proteins but not the apoplastic effector (Bas4) *in planta* [28]. In VdΔ*syn8* mutants, the retention of VdSCP10-GFP in the hyphopodium and inside the hyphal neck, suggests that the induction of SCP delivery in *V*. *dahliae* requires cues from the fungus-contacting surface and that VdΔ*syn8* mutants prevent the perception of information from the contact surface, and resulting in VdSCP10-GFP retention. Thus, effective delivery of secretory proteins during fungal infection in the host requires Syn8-mediated transport/cue-sensing via endosomes.

The final steps of the secretory pathway, which occur in the vicinity of the plasma membrane, are regulated by an array of small GTPases, the exocyst tethering complex, and SNARE proteins [20,30,44]. Co-localization of VdSec8-GFP and VdSep5-RFP and the absence of VdSep5 or VdExo70 to impair the delivery of secretory proteins to the penetration interface also support septin-dependent assembly of the exocyst in *V*. *dahliae*. Together with previous reports demonstrating that cytoplasmic effector accumulation in BICs of *M*. *oryzae* also required the exocyst components Exo70 and Sec5 [33], we assume that the effective delivery of secretory proteins during infection of plant hosts requires the exocyst coupled with SNARE proteins, such as Sec22 and Syn8, to tether vesicles loaded with secretory proteins to the plasma membrane.

**Fig 8:**
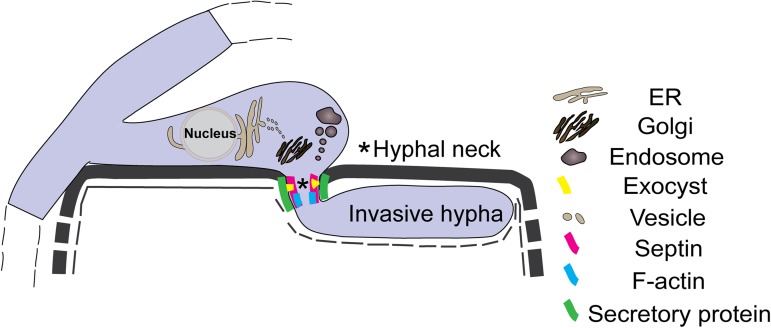
Simple schematic of secretory protein preferential delivery to the hyphal neck in *V*. *dahliae*. Simple schematic showing the accumulation/delivery of secretory proteins in the hyphal neck. A hyphopodium-mediated breach through the plant root cell wall and invasive hyphal growth. The penetration peg-derived hyphal neck joining the hyphopodium and the invasive hypha form the fungus-host penetration interface, where small secretory proteins are accumulated and secreted. The septin (VdSep5), F-actin, exocyst (VdExo70 and VdSec8), ER-Golgi traffic (VdSec22) and endosome-mediated traffic (VdSyn8) function in the delivery of secretory proteins to the penetration interface.

In summary, we provide evidence that hyphopodium-specific VdNoxB -regulated penetration peg formation accompanied by cytoskeletal organization of the septin-ring, form a fungus-host interface that functions as a site for the dynamic delivery of secretory proteins. The exocyst, VdSec22-mediated transport between the ER and Golgi apparatus and VdSyn8-mediated transport/cue-sensing via endosomes are involved in the secretion of secretory proteins, possibly including effectors, towards the interfaces (Fig 8). We assume that the fungal infectious structures function as key signaling hubs during plant infection and are the apparatus that not only breaches host cells but also generates unique interfaces for the secretion of fungal secretory proteins and associated regulatory components.

## Materials and methods

### Fungal isolates, culture conditions, infection assays and DNA analysis

The virulent defoliating *V*. *dahliae* isolate V592 from cotton that originated in Xinjiang, China, was used in this study. This isolate and its transformants were stored at –80°C and cultures were reactivated on potato dextrose agar (PDA) medium at 25°C in the dark. The conidia for the infection assays were cultured in liquid Czapek-Dox medium. Hyphae for microscopic observation were incubated on M0 medium with urea modified as NaNO_3_ [45].

For plant infection, cotton plants (‘Xinluzao No. 16’) were used in infection assays to evaluate the effect of *V*. *dahliae* isolate V592 and transformants on virulence using our laboratory’s unimpaired root-dip inoculation method, as described in our previous research [25]. Disease progression was recorded after 3 weeks of incubation. The infection assay for transformants was repeated three times. The symptoms were evaluated, and the disease grade was classified as follows: 0 (no symptoms), 1 (0–25% wilted leaves), 2 (25–50%), 3 (50–75%) and 4 (75–100%) [10]. The data were analyzed using the Student’s t-test.

Nucleic acid extraction and fungal transformation have all been previously described [25]. Single copy insertion was confirmed in transformants which were used to analyze the fluorescence intensity.

### Preparation of deletion constructs

To generate the knockout plasmids pKOVdSCP10, pKOVdSep3, pKOVdSep5, pKOVdSec22, pKOVdSyn8 and pKOVdExo70, upstream and downstream genomic sequences were amplified with the primers shown in S1 Table. The upstream and downstream genomic sequence pairs were inserted into a position flanking the hygromycin resistance cassette of vector pGKO-HPT with the USER enzyme to generate knock-out plasmids, and transformation was performed as previously described [46].

### Preparation of GFP fusion constructs

All of the GFP fusion constructs or RFP fusion constructs (next part) were generated by the infusion cloning method based on homologous recombination using the ClonExpress II or ClonExpress MultiS kit (Vazyme, China). The primers are shown in S1 Table. In each case, the primers contain a 15-20-bp overlap with adjoining fragments to allow the assembly of fragments by homologous recombination.

To select transformants with G418, the pSUL-NEO binary vector was created by insertion of the G418 resistance cassette amplified with Neo-F/R primers from pKOV21 into XbaI-digested pSULPH-GFP [25].

For convenient expression of the GFP fusion protein under the constitutive Tef promotor and TrpC terminator, we generated a binary vector pSUL-NEO-Tef-EKGFP-TrpC using the following steps: (1) a 0.7-kb 3GA-EGFP fragment, amplified with primers 3GAGFP-F (bearing three repeats of nucleotides encoding‘GA’) and GFP-Nt-R from pNPP9 [47], was cloned into the EcoRI/NotI sites of pNPP94 [48], resulting in pNPP94-3GAGFP; (2) we amplified the P*tef*:3GAGFP:T*trpc* fusion from pNPP94-3GAGFP using primers psul-ppn-HindIII-F and psul-ppn-EcoRI-R and recombined with the product HindIII/EcoRI-linearized pSUL-NEO to generate plasmid pSUL-NEO-Tef-3GAGFP-TrpC; (3) to separate GFP and the protein of interest, we introduced a linker with five repeats of nucleotides encoding the ‘EAAK’ motif [49] into EcoRI-digested pSUL-NEO-Tef-3GAGFP-TrpC, generating plasmid pSUL-NEO-Tef-EKGFP-TrpC.

To generate the P*tef*:VdSep5-GFP:T*trpc* construct, VdSep5 was amplified from the cDNA of V592 and fused into BamHI/EcoRI-linearized pSUL-NEO-Tef-EKGFP-TrpC.

To generate a binary vector that included the Tef promotor and TrpC terminator but lacked GFP, we amplified the Tef promoter and TrpC terminator from pNPP94 using primer psul-ppn-HindIII-F and psul-ppn-EcoRI-R and recombined the product with HindIII/EcoRI-linearized pSUL-NEOto generate pSUL-NEO-Tef-TrpC.

The P*noxb*:GFP-VdNoxB:T*trpc* construct was generated by cloning the GFP fragment between 2 kb upstream of the start codon and the genomic sequence of *VdNoxb*. The three sequences were fused into HindIII/EcoRI-linearized pSUL-NEO-Tef-TrpC.

To generate the of P*tef1*:LifeAct-GFP: T*trpc* fusion construct, we amplified P*tef1*: LifeAct-GFP from pAB261 [50] and integrated into HindIII/ EcoRI-linearized pSUL-NEO-Tef-TrpC.

To generate the C-terminal GFP fusion construct under the oliC promoter, the primer pair olic-HindIII-F and olic-BamHI-R were used to amplify the template pNAH-Grx1-roGFP2 plasmid [51], and the resulting PCR products were fused into HindIII/BamHI-digested pSUL-NEO-Tef-EKGFP-TrpC to generate pSUL-NEO-oliC-EKGFP-TrpC. To generate VdSCP8-GFP, VDSCP9-GFP, VDSCP10-GFP, VdSec8-GFP and VdExo70-GFP constructs with the oliC promoter, genomic sequences were amplified and fused into BamHI/EcoRI-linearized pSUL-NEO-oliC-EKGFP-TrpC.

To generate VdSCP8-GFP, VdSCP9-GFP, VdSCP10-GFP, VdSec8-GFP and VdExo70-GFP constructs with the native promoter, genomic sequences spanning 1.5–2 kb upstream of the start codon were amplified and fused into HindIII/EcoRI-linearized pSUL-NEO-Tef-EKGFP-TrpC using homologous recombination.

### Preparation of the RFP fusion construct

To co-express the RFP fusion construct with the GFP fusion construct, a nourseothricin resistance cassette was amplified using the primer pair nat-F/R with the pAL6-LifeAct plasmid as template [52] and fused into XbaI/XhoI-digested pSULPH-GFP to generate pNat-GFP. We amplified the Tef promoter and TrpC terminator from pNPP94 using the primers psul-ppn-HindIII-F and trpC-xbaI-R, and we recombined them with HindIII/EcoRI-linearized pNat-GFP to generate pNat-Tef-TrpC.

To study the localization relationship between VdSep5 and VdSCP10-GFP, we constructed P*tef*:VdSep5-RFP:T*trpc* which was selected by nourseothricin. First, we cloned the RFP sequence from plasmid pAL6-LifeAct [52] by EK-RFP-F and RFP-R into EcoRI-digested pNat-Tef-TrpC, resulting in pNat-Tef-EKRFP-TrpC. A VdSep5 cDNA fragment was fused into BamHI/EcoRI-digested pNat-Tef-EKRFP-TrpC, resulting in P*tef*:VdSep5-RFP:T*trpc* fusion. Transformants were selected in the presence of nourseothricin (50 μg/mL).

### Preparation of complementary constructs

For complementary VdΔ*sep3*, VdΔ*sec22* and Vd*Δsyn8* mutants, the corresponding genomic sequences, including 1.5–2 kb upstream of the start codon, were amplified with the primers listed in S1 Table and fused into HindIII/EcoRI-linearized pNat-Tef-TrpC by homologous recombination. Transformants were selected in the presence of nourseothricin (50 μg/mL). The P*tef*:VdSep5-GFP:T*trpc* construct and P*olic*:VdExo70-GFP:T*trpc* were used to complementVdΔ*sep5* and Vd*Δexo70* mutants, respectively.

### RNA extraction and cDNA

Total RNA was isolated from frozen mycelium collected from M0 medium cultured for 3d. *V*. *dahliae* cDNA was reverse-transcribed using SuperScript® III (Invitrogen).

### Quantitative real-time PCR

Before reverse transcription, residual DNA was removed from the total RNA using gDNA wiper (Vazyme). cDNA was reverse transcribed using HiScript II Q RT Supermix (Vazyme), and qRT-PCR was performed using ChamQ SYBR qPCR MasterMix (Vazyme) with the Bio-Rad CFX96 Real-Time system. The transcription levels of the target genes were quantified relative to the constitutively expressed elongation factor 1-α of *Verticillium dahliae* (VdElf). The gene-specific primers are listed in S1 Table. Biological replicates were performed three times.

### Light microscopy

To observe the infection of *V*. *dahliae*, *A*. *thaliana* roots were immersed in a conidial suspension (~10^5^ conidia/mL in water solution) for 10 min and then transferred onto a 0.75% agar plate at 25°C in the dark. To observe the protein localization of *V*. *dahliae* on cellophane, conidia were placed on cellophane and incubated at 25°C. The mycelium grown on cellophane for 3–9 days was used for protein localization assays. To compare the secretory difference between V592 and VdΔ*sec22*/VdΔ*syn8* mutants, the fungi were collected from the outer zone of the colony at the earliest time point for most V592 hyphal necks with ring signals. The fluorescence intensity data were analyzed using the Student’s t-test. Small pieces (~0.5 cm^2^) of cellophane with mycelium at the margin of the fungal colonies were cut with a scalpel and mounted in water. Images were obtained under a confocal laser microscope (Leica TCS SP8; Leica Microsystems) with a 100×oil immersion objective lens. The excitation wavelengths and emission filters were as follows: 488 nm/band-pass 500 to 550 nm for GFP, 561 nm/ band-pass 570 to 670 nm for RFP and FM4-64, and 405 nm/band-pass 400 to 600 nm for ER-Tracker. Confocal images were captured with a Leica hybrid detector and analyzed with Leica LAS AF software.

For each microscopy-based experiment, at least 20 images with three biological independent samples were observed for each micrograph to make conclusions. Each experiment was repeated at least twice.

### Transmission electron microscopy

For TEM observation, *V*. *dahliae*-infected cotton root and *V*. *dahliae* on cellophane were fixed immediately in 2.5% glutaraldehyde, buffered with PBS (pH 7.4) at 4°C overnight, washed with the same buffer four times and post-fixed with 1% osmium tetroxide for 1 h. Dehydration was then performed in an acetone series (50%, 75%, 85%, 95%, 100%), and the slices were embedded in Spurr’s resin mixture. Ultrathin serial sections (70 nm thickness) were cut from resin blocks, followed by uranyl acetate staining, and observed with a JEM-1400 electron microscope.

### Staining of fungi

For plasma membrane staining, FM4-64 (ThermoFisher) was used according to the manufacturer's protocol. For ER-Tracker staining, cultures were incubated at 30°C for 30 min with PBS containing 1 μM ER-Tracker™ Blue–White DPX (Molecular Probes) that had been pre-warmed at 30°C for 30 min, washed once with fresh PBS without the dye, and subjected to microscopic observation [53]. Next, 100 μg/mL FITC-conjugated wheat germ agglutinin (FITC-WGA, Sigma) was used to stain the fungal cell wall.

### Fluorescence recovery after photobleaching (FRAP) analysis

FRAP analyses was carried out with fungi on cellophane under a spinning disk confocal microscope (UltraVIEW VoX, Perkin Elmer, Beaconsfield, Buckinghamshire, UK) equipped with a Yokogawa Nipkow CSU-X1 spinning disk scanner, Hamamatsu EMCCD 9100–13, and Nikon TiE inverted microscope with the Perfect Focus System. We used the UltraVIEW PK Device to photobleach GFP. For the FRAP analyses, the specific region of interest (ROI) covering the entire fluorescence in the ring was selected for bleaching. Twenty bleaching iterations were performed using a 488 laser power of 60%. Image scans were obtained with 15% 488 laser power before and after bleaching. For quantitative analyses, the GFP fluorescence recovery curves were measured as the mean intensity of the ROI pixels, normalized using the using Volocity software (Perkin Elmer), and graphed using Microsoft Excel.

## Supporting information

S1 Fig Alignment of the predicted fungal amino acid sequences used in this study. **(A-F)** The *V*. *dahliae* amino acid sequences were aligned with each sequence of the putative homologs in *M*. *oryzae* (Mo) and *E*. *festucae* (Ef)/*S*. *cerevisiae* (Sc). Sequences were aligned using ClustalX2 and shaded using GeneDoc. Amino acid residues within a black background were identical among all of the listed proteins, gray residues were identical in two out of three of the listed proteins, and those shown on a white background did not show any similarity. VdSep3 (VDAG_00736) is aligned with *M*. *oryzae* (EHA54688.1) and *S*. *cerevisiae* (DAA09624.2) (A). VdSep5 (VDAG_04382) is aligned with *M*. *oryzae* (EHA45843.1) *and S*. *cerevisiae* (DAA08862.1) (B). VdSec22 (VDAG_08386) is aligned with *M*. *oryzae* (EHA47424.1) *and S*. *cerevisiae* (DAA09582.1) (C). VdSyn8 (VDAG_01236) is aligned with *M*. *oryzae* (EHA50711.1) *and S*. *cerevisiae* (DAA06974.1) (D). VdExo70 (VDAG_09051) is aligned with *M*. *oryzae* (EHA54952.1) *and S*. *cerevisiae* (DAA08714.1) (E). VdSec8 (VDAG_08435) is aligned with *M*. *oryzae* (EHA47501.1) *and S*. *cerevisiae* (DAA11477.1) (F).
(PDF)

S2 Fig Hyphopodium-specific VdNoxB-dependent development of the penetration peg in *V*. *dahliae*. (A) Hyphopodium-specific expression of GFP-tagged *V*. *dahliae* NADPH oxidase B (VdNoxB) under the native promoter (left) and localization of GFP-VdNoxB at the penetration peg (right). (B) Observation of the development of the VdNoxB-dependent hyphopodium and penetration peg in cellophane. Bar = 2.5 μm. (C) Observation of the penetration peg on cellophane at 3 dpi. The micrographs show two scanning layers of the upper side and base (below) of the hyphopodium. A thin penetration peg of wild-type V592 differentiated from the base of the hyphopodium and pierced the cellophane (below); VdΔ*noxb* developed a hyphopodium without the formation of a penetration peg.
(PDF)

S3 Fig Localization of the F-actin ring at the hyphal neck. (A-B) Micrographs of F-actin organization in the hyphal neck visualized by expression of LifeAct-GFP in V592. F-actin organized in the hyphal neck on cellophane (A) and on *Arabidopsis thaliana* root (B). Bar = 2.5μm.
(PDF)

S4 Fig Secretion of signal peptide containing proteins on the penetration interface. (A) Signal peptide analysis of VdSCP8, VdSCP9, VdSCP10 and VdIscI in *V*. *dahliae*. The signal peptide of each protein was predicted using the SignalP 4.1 server. The predicted signal peptides are marked in red color, and the 30 amino acids from the initiation codon are displayed. (B) Detection of the expression levels of selected SCPs by qRT-PCR. RNA samples isolated from 2-day-old fungal culture harvested in liquid Czapek-Dox (CD) medium, 4-day-old fungi cultured on cellophane and 2-day-old fungi on cotton roots. The relative expression levels were estimated using the 2^-ΔΔCt^ method. The expression level of each SCP gene in liquid culture was arbitrarily set to 1. The mean and standard errors were calculated from three independent replicates. The asterisks indicate significant differences (*P<0.05; Dunnett’s test). (C) VdSCP8-GFP expressed under the native promoter was detected at the penetration zone. The V592 transformant expressing VdSCP8-GFP under the native promoter was observed after growth on cellophane for 8 d. Bar = 2.5 μm. (D) The ring signals of SP_VdSCP8_-GFP, SP_VdSCP9_-GFP and SP_VdSCP10_-GFP at the penetration zone. *V*. *dahliae* transformants expressing SP_VdSCP8_-GFP, SP_VdSCP9_-GFP and SP_VdSCP10_-GFP under the control of the oliC promotor were used for the assay. The plasma membrane was stained with FM4-64 (red). Bar = 2.5 μm.
(PDF)

S5 Fig Targeted deletion of *VdSCP10* decreased virulence of *V*. *dahliae* in cotton plants. (A) Physical maps of the *VdSCP10* locus and the homologous recombination construct obtained by fusion of the *VdSCP10* 5′flack, hygromycin B resistance gene cassette and *VdSCP10* 3′flack. The probe and relative positions of primers used for PCR are indicated. (B) Southern blot analysis of targeted gene deletion mutants. EcoRI digested genomic DNA from the V592 wild type strain and two putative VdΔ*scp10* transformants were blotted with the probe indicated in the schematic diagram. (C) PCR amplification of genomic DNA from the complemented transformants using the primer pair in-F and in-R produced a banding pattern consistent with the integration of an intact gene in V592. (D) The colony morphology of the wild-type V592 and Vd*Δscp10* mutant strains and the corresponding complemented strains on PDA plates after a 2-week incubation. (E, F) Disease symptoms (E) and disease grades (F) of cotton plants infected with wild-type V592, Vd*Δscp10* mutant and the complementary strains at 21 dpi. The disease grade (DG) was calculated as previously described. Four is the highest DG, meaning that the entire plant died, while 0 is the lowest DG with no visible wilting. Three replicates of 36 plants were used for each inoculum (*P<0.05; t-test).
(PDF)

S6 Fig Targeted deletions of *VdSep3 and VdSep5* genes in *V*. *dahliae* strain V592. (A) Physical maps of the *VdSep3* locus and of the homologous recombination constructs obtained by fusion of the *VdSep3* 5′flack, hygromycin B resistance gene cassette and *VdSep3* 3′flack. Probes and relative positions of the primers used for PCR are indicated. *hph*, hygromycin resistance gene. (B) Southern blot analysis of targeted gene deletion mutants. BamHI digested genomic DNA from the V592 strain and two putative VdΔ*sep3* transformants were blotted with the probe indicated in the schematic diagram. KpnI and HindIII digested genomic DNA from V592 wild type strain and two putative VdΔ*sep5* transformants were analyzed as described above. (C) PCR amplification of genomic DNA from the complemented transformants using the primer pair in-F and in-R produced a banding pattern consistent with the integration of an intact *VdSep3* and *VdSep5*. Lanes 1–4 were using for the verification of Vd*Δsep3* complementation and lanes 5–8 were for the verification of Vd*Δsep5* complementation. (D) Colony morphology of V592, VdΔ*sep3* and VdΔ*sep5* mutants and the complementary strains on PDA plates incubated for 2 weeks. (E) Penetration ability analysis of Vd*Δsep3* and Vd*Δsep5*. Colonies of V592, Vd*Δsep3* and Vd*Δsep5* on M0 medium overlaid with cellophane (Before) and removal of the cellophane membrane (After). Images in the first row were obtained at 6 dpi, and the colonies below the cellophane were obtained at 9 dpi. (F) Hyphopodium morphology analysis of V592, VdΔ*sep3* and VdΔ*sep5*. images were obtained at 6 dpi. (G) Deficient development of the hyphopodium in Vd*Δsep3* and Vd*Δsep5*. Fungi incubated on cellophane at 5 dpi were used for the observation. The numbers of hyphopodia were counted in three fields of the culture under a light microscope at x1000 magnification with three replicates. The mean and SD for (G) were calculated from three clones for each mutant (*P<0.05; t-test). (H, I) Disease symptoms (H) and disease grades (I) of cotton plants infected with wild-type V592, Vd*Δsep3* and Vd*Δsep5* mutants and the complementary strains at 21 dpi. Three replicates of 36 plants were used for each inoculum. The asterisks indicate significant differences compared with V592 infection (*P<0.05; t-test).
(PDF)

S7 Fig Gene disruptions of *VdSec22*, *VdSyn8* and *VdExo70* in *V*. *dahliae*. (A) Physical maps of the *VdSec22* locus and the homologous recombination construct obtained by fusion of the *VdSec22* 5′flack, hygromycin B resistance gene cassette and *VdSec22* 3′flack. The probe and relative positions of primers used for PCR are indicated. The same approach was used for disruption of *VdSyn8* and *VdExo70*. (B) Southern blot analysis of targeted gene deletion mutants. NcoI-digested genomic DNA from V592 and two putative VdΔ*sec22* transformants were blotted with the probe indicated in the schematic diagram. NcoI-digested genomic DNA from the V592 and two putative VdΔ*syn8* transformants were analyzed as described above. SmaI and BamHI-digested genomic DNA from the V592 wild type strain and two putative VdΔ*exo70* transformants were analyzed as described above. (C) PCR amplification of genomic DNA from the complemented transformants using the primer pair in-F and in-R produced a banding pattern consistent with the integration of an intact gene in V592. Lanes 1–4, 5–8 and 9–12 were for the verification of VdΔ*syn8*, VdΔ*sec22* and VdΔ*exo70* complementation, respectively. (D) Colony morphology of wild-type V592 and Vd*Δexo70*, Vd*Δsec22* and Vd*Δsyn8* mutant strains and the corresponding complemented strains on PDA plates 2 weeks post-incubation.
(PDF)

S8 Fig Determination of the T-DNA insertional copy number of VdSCP10-GFP in V592, VdΔ*syn8*, VdΔ*sec22* and VdΔ*exo70*. Genomic DNA isolated from V592, VdΔ*syn8*, VdΔ*sec22* and VdΔ*exo70* expressing VdSCP10-GFP were digested with EcoRI for Southern blot analysis. Red arrowheads indicate selected colonies with single copy insertions for further study. Hybridization was performed with the ^32^P-labeled oliC promotor-specific DNA probe as shown below.
(PDF)

S9 Fig Deletion of *VdSec22* caused VdSCP10-GFP retention in the ER. VdSCP10-GFP expressed under the oliC promotor was transformed into V592 and VdΔ*sec22*. Staining the hyphal ER with ER-Tracker Blue-White DPX. Bar = 2.5μm.
(PDF)

S10 Fig Localization of *V*. *dahliae* exocyst subunits at the hyphal tips, hyphopodium base and hyphal neck. (A) Localization of VdSec8-GFP and VdExo70-GFP in the hyphal tips of *V*. *dahliae* on cellophane. (B) Localization of VdSec8-GFP and VdExo70-GFP at the base of the hyphopodium on cellophane. (C) VdSec8-GFP organized at the base of the hyphopodium on the surface of the root. (D) After invasive hyphae developed, VdSec8-GFP was organized at the hyphal neck on cellophane and plant roots. Bar = 2.5μm.
(PDF)

S11 Fig Colocalization of *V*. *dahliae* exocyst subunit VdSec8 and VdSep5 at the hyphal neck. Confocal laser scanning microscopy (CLSM) images and linescan graph showing co-localization of VdSec8-GFP and VdSep5-RFP at the hyphal neck. Bar = 2.5μm.
(PDF)

S1 Table Primers used in this study. (XLSX)

S1 Movie *Verticillium dahliae* colonization in cellophane. Confocal laser scanning microscopy (CLSM) images of hyphopodium development and penetration of *V*. *dahliae* V592 in cellophane. Fungal cell wall was stained with FITC-WGA. Movie was taken at 7dpi.
(AVI)

S2 Movie *Verticillium dahliae* colonization in *Arabidopsis thaliana* root. Confocal laser scanning microscopy (CLSM) images of hyphopodium development and penetration of *V*. *dahliae* V592 on *Arabidopsis thaliana* root. Fungal cell wall was stained with FITC-WGA. Movie was taken at 1 dpi.
(AVI)

## References

[1] Rep M (2005) Small proteins of plant-pathogenic fungi secreted during host colonization. FEMS Microbiol Lett 253: 19–27. 10.1016/j.femsle.2005.09.014 16216445

[2] Lyu X, Shen C, Fu Y, Xie J, Jiang D, et al (2016) A small secreted virulence-related protein is essential for the necrotrophic interactions of *Sclerotinia sclerotiorum* with its host plants. PLoS Pathog 12: e1005435 10.1371/journal.ppat.1005435 26828434 PMC4735494

[3] Klosterman SJ, Subbarao KV, Kang S, Veronese P, Gold SE, et al (2011) Comparative genomics yields insights into niche adaptation of plant vascular wilt pathogens. PLoS Pathog 7: e1002137 10.1371/journal.ppat.1002137 21829347 PMC3145793

[4] Kleemann J, Rincon-Rivera LJ, Takahara H, Neumann U, Ver Loren van Themaat E, et al (2012) Sequential delivery of host-induced virulence effectors by appressoria and intracellular hyphae of the phytopathogen *Colletotrichum higginsianum*. PLoS Pathog 8: e1002643 10.1371/journal.ppat.1002643 22496661 PMC3320591

[5] Irieda H, Maeda H, Akiyama K, Hagiwara A, Saitoh H, et al (2014) *Colletotrichum orbiculare* secretes virulence effectors to a biotrophic interface at the primary hyphal neck via exocytosis coupled with SEC22-mediated traffic. Plant Cell 26: 2265–2281. 10.1105/tpc.113.120600 24850852 PMC4079382

[6] Stergiopoulos I, de Wit PJ (2009) Fungal effector proteins. Annu Rev Phytopathol 47: 233–263. 10.1146/annurev.phyto.112408.132637 19400631

[7] Khang CH, Berruyer R, Giraldo MC, Kankanala P, Park SY, et al (2010) Translocation of *Magnaporthe oryzae* effectors into rice cells and their subsequent cell-to-cell movement. Plant Cell 22: 1388–1403. 10.1105/tpc.109.069666 20435900 PMC2879738

[8] Mosquera G, Giraldo MC, Khang CH, Coughlan S, Valent B (2009) Interaction Transcriptome Analysis Identifies Magnaporthe oryzae BAS1-4 as Biotrophy-Associated Secreted Proteins in Rice Blast Disease. The Plant Cell 21: 1273–1290. 10.1105/tpc.107.055228 . 19357089 PMC2685627

[9] de Jonge R, van Esse HP, Maruthachalam K, Bolton MD, Santhanam P, et al (2012) Tomato immune receptor Ve1 recognizes effector of multiple fungal pathogens uncovered by genome and RNA sequencing. Proc Natl Acad Sci U S A 109: 5110–5115. 10.1073/pnas.1119623109 22416119 PMC3323992

[10] Liu T, Song T, Zhang X, Yuan H, Su L, et al (2014) Unconventionally secreted effectors of two filamentous pathogens target plant salicylate biosynthesis. Nat Commun 5: 4686 10.1038/ncomms5686 25156390 PMC4348438

[11] Dou D, Kale SD, Wang X, Jiang RH, Bruce NA, et al (2008) RXLR-mediated entry of Phytophthora sojae effector Avr1b into soybean cells does not require pathogen-encoded machinery. Plant Cell 20: 1930–1947. 10.1105/tpc.107.056093 18621946 PMC2518231

[12] Kale SD, Tyler BM (2011) Entry of oomycete and fungal effectors into plant and animal host cells. Cellular Microbiology 13: 1839–1848. 10.1111/j.1462-5822.2011.01659.x 21819515

[13] Fradin EF, Thomma BP (2006) Physiology and molecular aspects of *Verticillium* wilt diseases caused by *V*. *dahliae* and *V*. *albo-atrum*. Mol Plant Pathol 7: 71–86. 10.1111/j.1364-3703.2006.00323.x 20507429

[14] Klosterman SJ, Atallah ZK, Vallad GE, Subbarao KV (2009) Diversity, pathogenicity, and management of *Verticillium* species. Annu Rev Phytopathol 47: 39–62. 10.1146/annurev-phyto-080508-081748 19385730

[15] Inderbitzin P, Subbarao KV (2014) *Verticillium* systematics and evolution: how confusion impedes *Verticillium* wilt management and how to resolve it. Phytopathology 104: 564–574. 10.1094/PHYTO-11-13-0315-IA 24548214

[16] Zhao YL, Zhou TT, Guo HS (2016) Hyphopodium-specific VdNoxB/VdPls1-dependent ROS-Ca^2+^ signaling is required for plant infection by *Verticillium dahliae*. PLoS Pathog 12: e1005793 10.1371/journal.ppat.1005793 27463643 PMC4962994

[17] Ryder LS, Dagdas YF, Mentlak TA, Kershaw MJ, Thornton CR, et al (2013) NADPH oxidases regulate septin-mediated cytoskeletal remodeling during plant infection by the rice blast fungus. Proc Natl Acad Sci U S A 110: 3179–3184. 10.1073/pnas.1217470110 23382235 PMC3581893

[18] Dagdas YF, Yoshino K, Dagdas G, Ryder LS, Bielska E, et al (2012) Septin-mediated plant cell invasion by the rice blast fungus, *Magnaporthe oryzae*. Science 336: 1590–1595. 10.1126/science.1222934 22723425

[19] Mavrakis M, Azou-Gros Y, Tsai FC, Alvarado J, Bertin A, et al (2014) Septins promote F-actin ring formation by crosslinking actin filaments into curved bundles. Nature Cell Biology 16: 322–334. 10.1038/ncb2921 24633326

[20] Gupta YK, Dagdas YF, Martinez-Rocha AL, Kershaw MJ, Littlejohn GR, et al (2015) Septin-dependent assembly of the exocyst is essential for plant infection by *Magnaporthe oryzae*. Plant Cell 27: 3277–3289. 10.1105/tpc.15.00552 26566920 PMC4682301

[21] Petersen TN, Brunak S, von Heijne G, Nielsen H (2011) SignalP 4.0: discriminating signal peptides from transmembrane regions. Nat Methods 8: 785–786. 10.1038/nmeth.1701 21959131

[22] de Jonge R, Thomma BP (2009) Fungal LysM effectors: extinguishers of host immunity? Trends Microbiol 17: 151–157. 10.1016/j.tim.2009.01.002 19299132

[23] de Jonge R, van Esse HP, Kombrink A, Shinya T, Desaki Y, et al (2010) Conserved fungal LysM effector Ecp6 prevents chitin-triggered immunity in plants. Science 329: 953–955. 10.1126/science.1190859 20724636

[24] Kombrink A, Rovenich H, Shi-Kunne X, Rojas-Padilla E, van den Berg GC, et al (2016) Verticillium dahliae LysM effectors differentially contribute to virulence on plant hosts. Mol Plant Pathol. 10.1111/mpp.12520 PMC6638240 27911046

[25] Gao F, Zhou BJ, Li GY, Jia PS, Li H, et al (2010) A glutamic acid-rich protein identified in *Verticillium dahliae* from an insertional mutagenesis affects microsclerotial formation and pathogenicity. PLoS One 5: e15319 10.1371/journal.pone.0015319 21151869 PMC2998422

[26] Lewis MJ, Pelham HR (2002) A new yeast endosomal SNARE related to mammalian syntaxin 8. Traffic 3: 922–929. 12453154 10.1034/j.1600-0854.2002.31207.x

[27] Jahn R, Scheller RH (2006) SNAREs—engines for membrane fusion. Nature Reviews Molecular Cell Biology 7: 631–643. 10.1038/nrm2002 16912714

[28] Qi Z, Liu M, Dong Y, Zhu Q, Li L, et al (2016) The syntaxin protein (MoSyn8) mediates intracellular trafficking to regulate conidiogenesis and pathogenicity of rice blast fungus. New Phytol 209: 1655–1667. 10.1111/nph.13710 26522477

[29] Lewis MJ, Rayner JC, Pelham HR (1997) A novel SNARE complex implicated in vesicle fusion with the endoplasmic reticulum. EMBO J 16: 3017–3024. 10.1093/emboj/16.11.3017 9214619 PMC1169920

[30] Synek L, Sekeres J, Zarsky V (2014) The exocyst at the interface between cytoskeleton and membranes in eukaryotic cells. Frontiers in Plant Science 4: 543 10.3389/fpls.2013.00543 24427163 PMC3877765

[31] TerBush DR, Maurice T, Roth D, Novick P (1996) The Exocyst is a multiprotein complex required for exocytosis in Saccharomyces cerevisiae. EMBO J 15: 6483–6494. 8978675 PMC452473

[32] Kee Y, Yoo JS, Hazuka CD, Peterson KE, Hsu SC, et al (1997) Subunit structure of the mammalian exocyst complex. Proc Natl Acad Sci U S A 94: 14438–14443. 9405631 10.1073/pnas.94.26.14438 PMC25013

[33] Giraldo MC, Dagdas YF, Gupta YK, Mentlak TA, Yi M, et al (2013) Two distinct secretion systems facilitate tissue invasion by the rice blast fungus *Magnaporthe oryzae*. Nat Commun 4: 1996 10.1038/ncomms2996 23774898 PMC3709508

[34] Tucker SL, Besi MI, Galhano R, Franceschetti M, Goetz S, et al (2010) Common genetic pathways regulate organ-specific infection-related development in the rice blast fungus. Plant Cell 22: 953–972. 10.1105/tpc.109.066340 20348434 PMC2861474

[35] Ryder LS, Talbot NJ (2015) Regulation of appressorium development in pathogenic fungi. Curr Opin Plant Biol 26: 8–13. 10.1016/j.pbi.2015.05.013 26043436 PMC4781897

[36] Dobbelaere J, Gentry MS, Hallberg RL, Barral Y (2003) Phosphorylation-dependent regulation of septin dynamics during the cell cycle. Dev Cell 4: 345–357. 12636916 10.1016/s1534-5807(03)00061-3

[37] Vargas-Muñiz JM, Renshaw H, Richards AD, Waitt G, Soderblom EJ, et al (2016) Dephosphorylation of the Core Septin, AspB, in a Protein Phosphatase 2A-Dependent Manner Impacts Its Localization and Function in the Fungal Pathogen Aspergillus fumigatus. Frontiers in Microbiology 7:997 10.3389/fmicb.2016.00997 27446037 PMC4916205

[38] Low IC, Loh T, Huang Y, Virshup DM, Pervaiz S (2014) Ser70 phosphorylation of Bcl-2 by selective tyrosine nitration of PP2A-B56delta stabilizes its antiapoptotic activity. Blood 124: 2223–2234. 10.1182/blood-2014-03-563296 25082878

[39] Bourett TM, Howard RJ (1990) In vitro development of penetration structures in the rice blast fungus Magnaporthe grisea. Canadian Journal of Botany 68: 329–342.

[40] Cheng Q, Wang H, Xu B, Zhu S, Hu L, et al (2014) Discovery of a novel small secreted protein family with conserved N-terminal IGY motif in Dikarya fungi. BMC Genomics 15: 1151 10.1186/1471-2164-15-1151 25526808 PMC4367982

[41] Song W, Dou X, Qi Z, Wang Q, Zhang X, et al (2010) R-SNARE homolog MoSec22 is required for conidiogenesis, cell wall integrity, and pathogenesis of *Magnaporthe oryzae*. PLoS One 5: e13193 10.1371/journal.pone.0013193 20949084 PMC2950850

[42] Haridas S, Wang Y, Lim L, Alamouti SM, Jackman S, et al (2013) The genome and transcriptome of the pine saprophyte *Ophiostoma piceae*, and a comparison with the bark beetle-associated pine pathogen *Grosmannia clavigera*. BMC Genomics 14: 373 10.1186/1471-2164-14-373 23725015 PMC3680317

[43] Bielska E, Higuchi Y, Schuster M, Steinberg N, Kilaru S, et al (2014) Long-distance endosome trafficking drives fungal effector production during plant infection. Nat Commun 5: 5097 10.1038/ncomms6097 25283249 PMC4205857

[44] Taheri-Talesh N, Horio T, Araujo-Bazan L, Dou X, Espeso EA, et al (2008) The tip growth apparatus of *Aspergillus nidulans*. Mol Biol Cell 19: 1439–1449. 10.1091/mbc.E07-05-0464 18216285 PMC2291424

[45] Brun S, Malagnac F, Bidard F, Lalucque H, Silar P (2009) Functions and regulation of the Nox family in the filamentous fungus Podospora anserina: a new role in cellulose degradation. Mol Microbiol 74: 480–496. 10.1111/j.1365-2958.2009.06878.x 19775249

[46] Wang S, Xing HY, Hua CL, Guo HS, Zhang J (2016) An improved single-step cloning strategy simplifies the *Agrobacterium tumefaciens*-mediated transformation (ATMT)-based gene-disruption eethod for *Verticillium dahliae*. Phytopathology 106: 645–652. 10.1094/PHYTO-10-15-0280-R 26780432

[47] Takemoto D, Kamakura S, Saikia S, Becker Y, Wrenn R, et al (2011) Polarity proteins Bem1 and Cdc24 are components of the filamentous fungal NADPH oxidase complex. Proc Natl Acad Sci U S A 108: 2861–2866. 10.1073/pnas.1017309108 21282602 PMC3041104

[48] Takemoto D, Tanaka A, Scott B (2006) A p67Phox-like regulator is recruited to control hyphal branching in a fungal-grass mutualistic symbiosis. Plant Cell 18: 2807–2821. 10.1105/tpc.106.046169 17041146 PMC1626622

[49] Arai R, Ueda H, Kitayama A, Kamiya N, Nagamune T (2001) Design of the linkers which effectively separate domains of a bifunctional fusion protein. Protein Engineering 14: 529–532. 11579220 10.1093/protein/14.8.529

[50] Berepiki A, Lichius A, Shoji JY, Tilsner J, Read ND (2010) F-actin dynamics in *Neurospora crassa*. Eukaryot Cell 9: 547–557. 10.1128/EC.00253-09 20139238 PMC2863416

[51] Heller J, Meyer AJ, Tudzynski P (2012) Redox-sensitive GFP2: use of the genetically encoded biosensor of the redox status in the filamentous fungus *Botrytis cinerea*. Mol Plant Pathol 13: 935–947. 10.1111/j.1364-3703.2012.00802.x 22524254 PMC6638776

[52] Lichius AR N. (2011) A versatile set of Lifeact-RFP expression plasmids for live-cell imaging of F-actin in filamentous fungi. Fungal Genet Rep 57: 8–14.

[53] Kuratsu M, Taura A, Shoji JY, Kikuchi S, Arioka M, et al (2007) Systematic analysis of SNARE localization in the filamentous fungus *Aspergillus oryzae*. Fungal Genet Biol 44: 1310–1323. 10.1016/j.fgb.2007.04.012 17590362

