# Supplementary material for: Secretory proteins are delivered to the septin-organized penetration interface during root infection by Verticillium dahliae
Source: PLoS Pathog. 2017 Mar 10;13(3):e1006275. doi: 10.1371/journal.ppat.1006275 (PMC5362242; doi:10.1371/journal.ppat.1006275)
Supplement: S1 Fig — (A-F) The V. dahliae amino acid sequences were aligned with each sequence of the putative homologs in M. oryzae (Mo) and E. festucae (Ef)/S. cerevisiae (Sc). Sequences were aligned using ClustalX2 and shaded using GeneDoc. Amino acid residues within a black background were identical among all of the listed proteins, gray residues were identical in two out of three of the listed proteins, and those shown on a white background did not show any similarity. VdSep3 (VDAG_00736) is aligned with M. oryzae (EHA54688.1) and S. cerevisiae (DAA09624.2) (A). VdSep5 (VDAG_04382) is aligned with M. oryzae (EHA45843.1) and S. cerevisiae (DAA08862.1) (B). VdSec22 (VDAG_08386) is aligned with M. oryzae (EHA47424.1) and S. cerevisiae (DAA09582.1) (C). VdSyn8 (VDAG_01236) is aligned with M. oryzae (EHA50711.1) and S. cerevisiae (DAA06974.1) (D). VdExo70 (VDAG_09051) is aligned with M. oryzae (EHA54952.1) and S. cerevisiae (DAA08714.1) (E). VdSec8 (VDAG_08435) is aligned with M. oryzae (EHA47501.1) and S. cerevisiae (DAA11477.1) (F). (PDF) [file ppat.1006275.s001.pdf]

Supplementary Figures

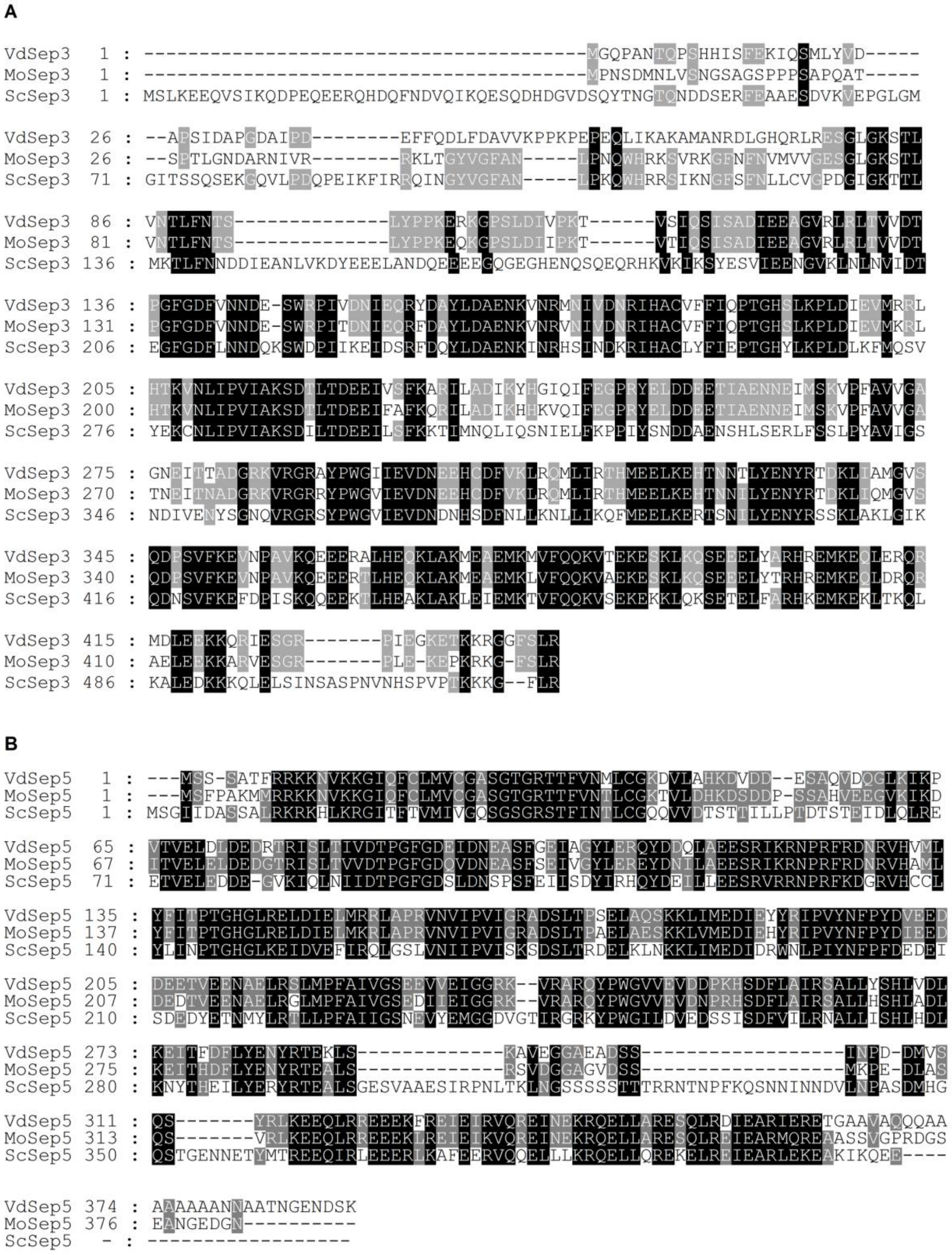

C

```
VdSec22   1 : MKSIRSTQIARLDGLMLCASVDDEHQEGDVVEIKNQIRAVLRKVTRKSEPRASLELGQA-TLNYLIENDI
MoSec22   1 : --MIRSTQIARLDGLMLCASVDEDASDSALAEVKSQVKLVLRRLNRNSETQASIESGSAYTLHYLIAGDV
ScSec22   1 : --MIKSTLIYREDGLPLCTSVDNEN-DPSLFEQKQKVKIVVSRLTPQSATEATLESGSF-EIHYLKKSMV

VdSec22  70 : IFLTITDRQYPRNLTFTYLSDLATEFQKTYPPAQLQSPTLRPYAFMEFDTFITRTKATYSDARAANNLDK
MoSec22  69 : VYVCISDRSYPRKLAFTYLSDLATEFANTYSQAQVTSPNLRPYAFVEFDTFIGRTRATYTDARATQNLDK
ScSec22  67 : YYFVICESGYPRNLAFSYLNDIAQEFEHSFAN-EYPKPTVRPYQFVNFDNFLQMTKKSYSDKKVQDNLDQ

VdSec22 140 : LNDELRDVTKVMTKNIEDLLYRGDSLERMGELSSRLRDDSKKYKRAAVRINWELLLKQYGPIGGLGLFII
MoSec22 139 : LNDELRDVTKVMTKNIEDLLYRGDSLERMGELSSRLRDDSKKYRRAAVRINWELMLKQYGPFAGLGAFIL
ScSec22 136 : LNQELVGVKQIMSKNIEDLLYRGDSLDKMSDMSSSLKETSKRYRKSAQKINFDLLISQYAPIVIVAFFFV

VdSec22 210 : FFLWWRFF-
MoSec22 209 : FFLWWRFF-
ScSec22 206 : FLFWWIFLK
```

D

```
VdSyn8   1 : MSKPNQLFLLADHIKLSLLERQRAKSLNLDDDSQEGHISRSLDQFRDGLEALQEEQRRQTESGDASGAQS
MoSyn8   1 : MSNQNALFLLADHIKLSLLERKRAQSLNVDAGSQDGHISRSLDQFREGLEALEKEQKRSEEAGDEDKALD
ScSyn8   1 : ---MDVLKLGYELDQLSDLVEERTRLVSVLKLAPTSNDNVTLKRQLGSILELLQKCAPNDE---------

VdSyn8  71 : IADALPALQKQFSDLTSQFHGFPTPETTSTLTQPNDPSLSPDFAHAQSATAATAGPPRKTTLRSPSSSST
MoSyn8  71 : ISDSLPSLQKQLDDLTSQFHGFSSVAPST----PNDPSLASDFAAAESVPAAGS---RAKQVR-FSDSPM
ScSyn8  59 : -------LISRYNTILDKIP---------------DTAVDKELYRFQQQVARNTDEVSKESLKKVRFKND

VdSyn8 141 : IPKTVRFTDSAPSPSAADPALTAELFGPYRDEP-SETEHRDATAGAG---LDNQQIHAYHARVLREQDDH
MoSyn8 133 : AAGSRRGNDDE----DLEAQRSGLLGQPYRDDPDSGPTFRDQIEEQG---LDNVQVHAYHQRVMEEQDAQ
ScSyn8 107 : DELTVMYKDDD---EQDEESPLPSTHTPYKDEPLQSQLQSQSQPQPPQPMVSNQELFINQQQQLLEQDSH

VdSyn8 207 : LDRLGESIGRQRELSMQIGDELDS-HVAMLDDVDAATDRHQGRLDRARRNLNKVARS-AGESKQMMTIIA
MoSyn8 196 : LDALGASISRQRELSMQIGDELDS-QVAMLDESERVVDRHQSSLDRARRQVGRISRS-AGETKQFGVIIA
ScSyn8 174 : LGALSQSIGRTHDISLDLNNEIVSQNDSLLVDLENLIDNNGRNLNRASRSMHGFNNSRFKDNGNCVIILV

VdSyn8 275 : LIIILVLLIAVLK
MoSyn8 264 : LIVILVLLIAIFK
ScSyn8 244 : LIVVLLLLLVL-
```

E

```
VdExo70   1 : MAVTVTNNRQIADEEARAEVDVLSSRLEKTTQLTRKIQASLGRLDATGKSVRDVAGPLNGETKKLQVLGH
MoExo70   1 : MAVGLANGRHAAEEEARAEVDVLNSRLEKTTQLTKKIEACLVRLESTGKSVREVAGPLNGETKKLQVLGH
ScExo70   1 : --------MPAEIDIDEADVLVLSQELQKTSKLTFEINKSLKKIAATSNQSSQLFTPILARNNVLTTLQR

VdExo70  71 : NIDKVVSSIEKLRQPADSKNDEEQIIRAGPDKAGLVNYLASVKRLNQALADMQTSNLRANQQT-----IA
MoExo70  71 : NIDNVLSAIERLRAPADSKNDEEQIIRMGPEKADLPNYLNSLKRLNKALVDMKASNLRSNQQT-----MN
ScExo70  63 : NIESTLNSVASVKDLANEASKYEIILQKGINQVGLKQYTQVVHKLDDMLEDIQSGQANREENSEFHGILT

VdExo70 136 : DLSRLVKSGNTQLEGHFDKLIRAETPRSIEPLHYITKDVPFPTLAQSVITPLALIRAHVAG--RQPQLTS
MoExo70 136 : DLQRLVTLGTTQLATLFDKLLRSETPRSLEPLHYITKDKPFPLLSRDNVNRLGPIYSFVAGSNRQGKGVG
ScExo70 133 : HLEQLIKRSEAQLRVYFISILNSIKP--FDPQINITKKMPFPYYEDQQLGALSWILDYFHG-------NS

VdExo70 204 : QESPAAKIFADVRGQYLSSTLVNLAAASVNTAKKKNSEAIYRAGTNGIGTYAQAMEGLFLAEYENICNLF
MoExo70 206 : SESTIAEVYSEVRGPYLAETLANLAAASVNTAKKKNPDAVYRAGTNGIGTYAQAMERLFQAEYENITRIF
ScExo70 194 : EGSIIQDILVGERSKLILKCMAFLEPFAKEISTAKN--APYEKGSSGMNSYTEALLGFIANEKSLVDDLY

VdExo70 274 : NR--GDWGAIFQVTCQAALAELARAVRELNVHIKAHLTTDCYLAYEITEIMSSLSEKLETRTGELKSSLA
MoExo70 276 : SR--EDWAPLFQATCQNAIVELSRTLREINAHIKVHLNTDCFLAYEIVEIISGMSSRLED----LRAAFA
ScExo70 262 : SQYTESKPHVLSQILSPLISAYAKLFGANLKIVRSNLENFGFFSFELVESINDVKKSLRGKELQNYNLLQ

VdExo70 342 : AALRPVRETAKSSLADLLEDTKRRVGNMQVLPADGAPVPIVSETMQRLQAMVEFMRPISSIMVSLGDGGW
MoExo70 340 : ACLKPVRETAKTSLGELIEDTKRKVANMQSIPADGAPSPVIAETMQRLQTMVEFLRPVSSIMISIGNGGW
ScExo70 332 : DCTQEVRQVTQSLFRDAIDRIIKKANSISTIPSNNGVTEATVDTMSRLRKFSEYKNGCLGAMDNITRENW

VdExo70 412 : KSVAAANGRSADVIPSLASFDIGADGKDIFSHYCTDTIETLLSSLNQKAQAVLRGG--------------
MoExo70 410 : KSLASSRG-GGDALPSLASFDVGANGQEIFADYCSDTIDILLLSLDGKAR-MMNGK--------------
ScExo70 402 : LPSNYKEKEYTLQNEALN----WEDHNVLLSCFISDCIDTLAVNLERKAQIALMPNQEPDVANPNSSKNK

VdExo70 468 : -RGVLGVFLANNVVIVERMIRDSDLAPLLEQR-LALLDQWRKKSKSLYTMDCKEVSSHLFDVIHTNK-QR
MoExo70 464 : -KPVVGVFIANSIAITERSIRESDLAPLMETR-LGILETYRKKAKLYYTEPCKDVSMHLFDVIHTSKSAR
ScExo70 468 : HKQRIGFFILMNLTLVEQIVEKSELNLMLAGEGHSRLERLKKRYISYMVSDWRDLTANLMDSVFIDS---

VdExo70 535 : PTSGQP--DSSSIVKSLSSKDKDNIKNKFQSFNASFDEMVAKHKSYNMER-EVRQMFARDIQQMLEPLYN
MoExo70 532 : PSSGQASADSATILKQLSSKDKESIKNKFTSFNAAFDDMVARHKSFSMER-EVRQMFARDMQQMLEPLYV
ScExo70 535 : --SGKK------------SKDKEQIKEKFRKFNEGFEDLVSKTKQYKLSDPSLKVTLKSEIISLVMPMYE

VdExo70 602 : RFWDRYHEVDKGKGKYVKYDKSAISAIFVTLY-
MoExo70 601 : RFWDRYHEVDKGKGKYVKYDKAAIAAVFASLY-
ScExo70 591 : RFYSRYKDSFKNPRKHIKYTPDELTTVLNQLVR
```

F

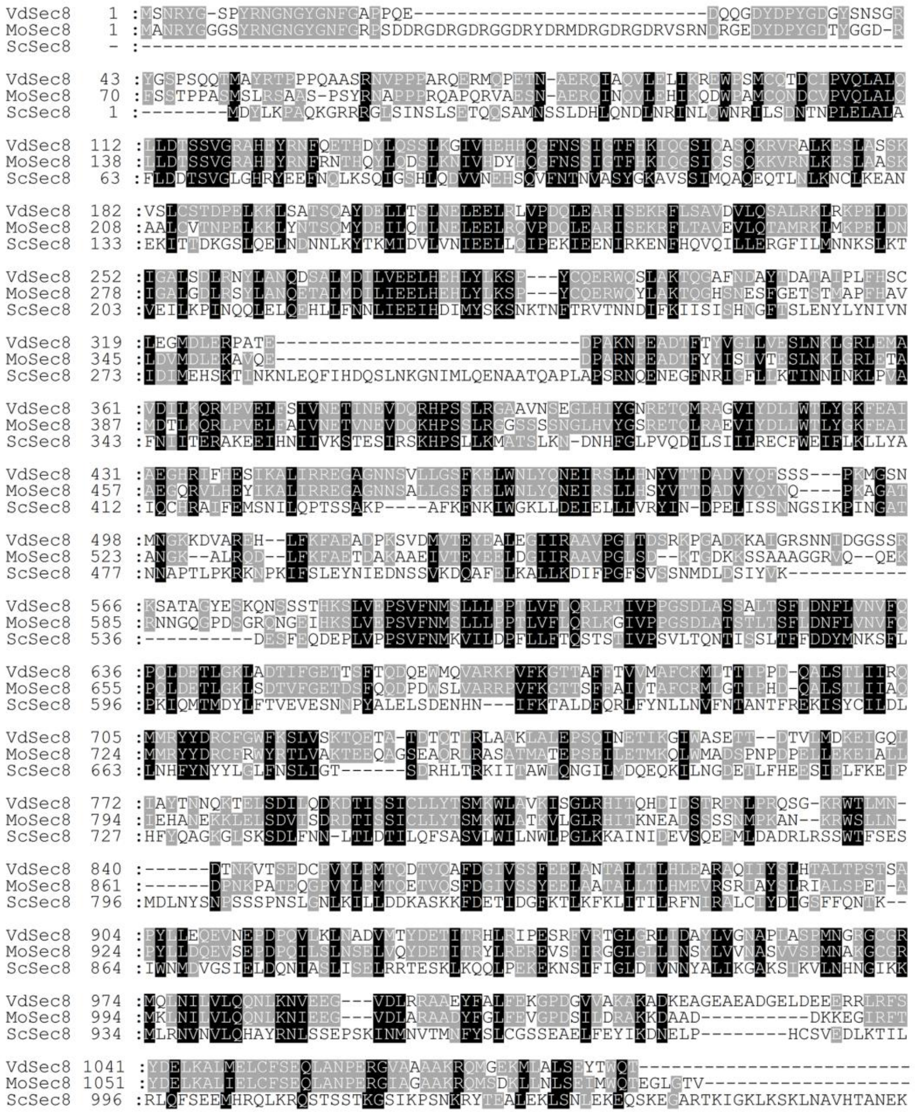

S1 Fig. Alignment of the predicted fungal amino acid sequences used in this study. (A-F) The *V. dahliae* amino acid sequences were aligned with each sequence of the putative homologs in *M. oryzae* (Mo) and *E. festucae* (Ef)/*S. cerevisiae* (Sc). Sequences were aligned using ClustalX2 and shaded using GeneDoc. Amino acid residues within a black background were identical among all of the listed proteins, gray residues were identical in two out of three of the listed proteins, and those shown on a white background did not show any similarity. VdSep3 (VDAG_00736) is aligned with *M. oryzae* (EHA54688.1) and *S. cerevisiae* (DAA09624.2) (A). VdSep5 (VDAG_04382) is aligned with *M. oryzae* (EHA45843.1) *and S. cerevisiae* (DAA08862.1) (B). VdSec22 (VDAG_08386) is aligned with *M. oryzae* (EHA47424.1) *and S. cerevisiae* (DAA09582.1) (C). VdSyn8 (VDAG_01236) is aligned with *M. oryzae* (EHA50711.1) *and S. cerevisiae* (DAA06974.1) (D). VdExo70 (VDAG_09051) is aligned with *M. oryzae* (EHA54952.1) *and S. cerevisiae* (DAA08714.1) (E). VdSec8 (VDAG_08435) is aligned with *M. oryzae* (EHA47501.1) *and S. cerevisiae* (DAA11477.1) (F).
