# Supplementary material for: Secretory proteins are delivered to the septin-organized penetration interface during root infection by Verticillium dahliae
Source: PLoS Pathog. 2017 Mar 10;13(3):e1006275. doi: 10.1371/journal.ppat.1006275 (PMC5362242; doi:10.1371/journal.ppat.1006275)
Supplement: S2 Fig — (A) Hyphopodium-specific expression of GFP-tagged V. dahliae NADPH oxidase B (VdNoxB) under the native promoter (left) and localization of GFP-VdNoxB at the penetration peg (right). (B) Observation of the development of the VdNoxB-dependent hyphopodium and penetration peg in cellophane. Bar = 2.5 μm. (C) Observation of the penetration peg on cellophane at 3 dpi. The micrographs show two scanning layers of the upper side and base (below) of the hyphopodium. A thin penetration peg of wild-type V592 differentiated from the base of the hyphopodium and pierced the cellophane (below); VdΔnoxb developed a hyphopodium without the formation of a penetration peg. (PDF) [file ppat.1006275.s002.pdf]

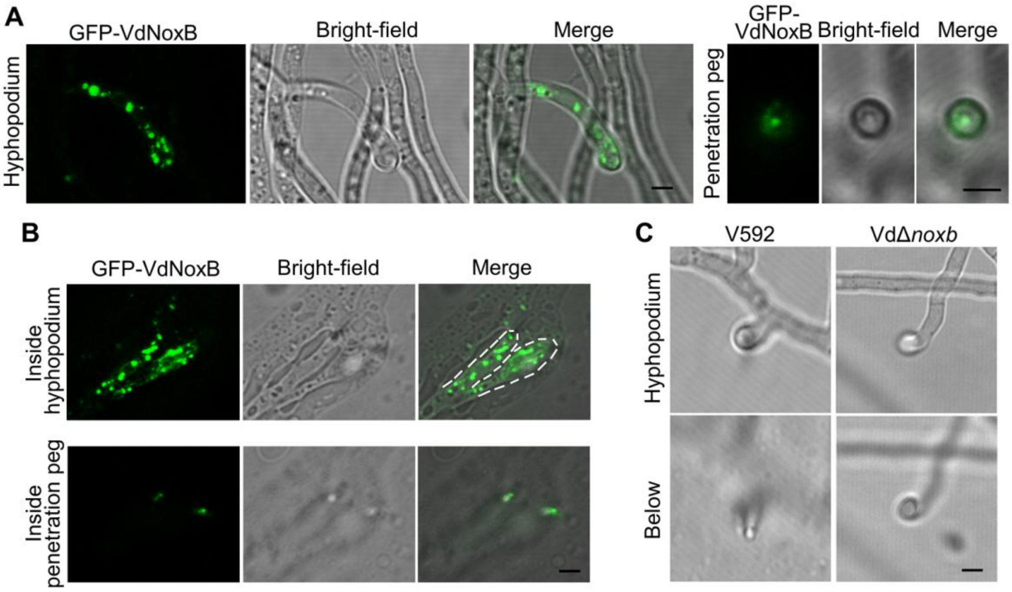

S2 Fig. Hyphopodium-specific VdNoxB-dependent development of the penetration peg in *V. dahliae.*

(A) Hyphopodium-specific expression of GFP-tagged *V. dahliae* NADPH oxidase B (VdNoxB) under the native promoter (left) and localization of GFP-VdNoxB at the penetration peg (right). (B) Observation of the development of the VdNoxB-dependent hyphopodium and penetration peg in cellophane. Bar = 2.5 μm. (C) Observation of the penetration peg on cellophane at 3 dpi. The micrographs show two scanning layers of the upper side and base (below) of the hyphopodium. A thin penetration peg of wild-type V592 differentiated from the base of the hyphopodium and pierced the cellophane (below); VdΔ*noxb* developed a hyphopodium without the formation of a penetration peg.
