# Supplementary material for: Secretory proteins are delivered to the septin-organized penetration interface during root infection by Verticillium dahliae
Source: PLoS Pathog. 2017 Mar 10;13(3):e1006275. doi: 10.1371/journal.ppat.1006275 (PMC5362242; doi:10.1371/journal.ppat.1006275)
Supplement: S3 Fig — (A-B) Micrographs of F-actin organization in the hyphal neck visualized by expression of LifeAct-GFP in V592. F-actin organized in the hyphal neck on cellophane (A) and on Arabidopsis thaliana root (B). Bar = 2.5μm. (PDF) [file ppat.1006275.s003.pdf]

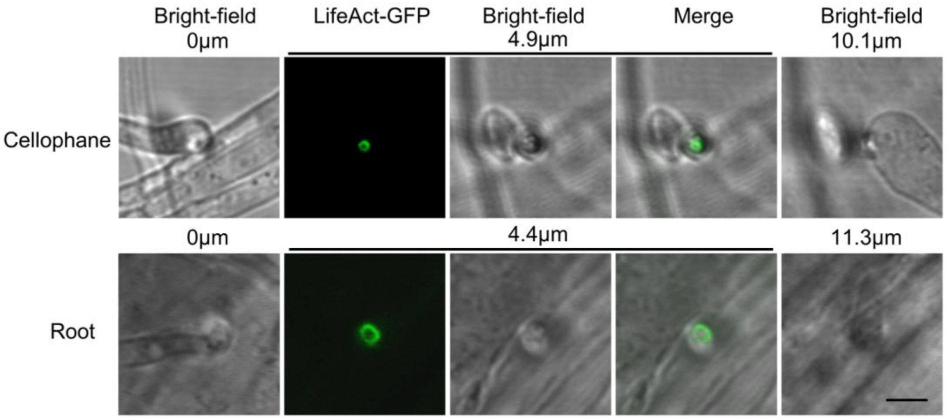

S3 Fig. Localization of the F-actin ring at the hyphal neck.

Micrographs of F-actin organization in the hyphal neck visualized by expression of LifeAct-GFP in V592. F-actin organized in the hyphal neck on cellophane and on *Arabidopsis thaliana* root. Bar = 2.5 µm.
