# Supplementary material for: Secretory proteins are delivered to the septin-organized penetration interface during root infection by Verticillium dahliae
Source: PLoS Pathog. 2017 Mar 10;13(3):e1006275. doi: 10.1371/journal.ppat.1006275 (PMC5362242; doi:10.1371/journal.ppat.1006275)
Supplement: S4 Fig — (A) Signal peptide analysis of VdSCP8, VdSCP9, VdSCP10 and VdIscI in V. dahliae. The signal peptide of each protein was predicted using the SignalP 4.1 server. The predicted signal peptides are marked in red color, and the 30 amino acids from the initiation codon are displayed. (B) Detection of the expression levels of selected SCPs by qRT-PCR. RNA samples isolated from 2-day-old fungal culture harvested in liquid Czapek-Dox (CD) medium, 4-day-old fungi cultured on cellophane and 2-day-old fungi on cotton roots. The relative expression levels were estimated using the 2-ΔΔCt method. The expression level of each SCP gene in liquid culture was arbitrarily set to 1. The mean and standard errors were calculated from three independent replicates. The asterisks indicate significant differences (*P<0.05; Dunnett’s test). (C) VdSCP8-GFP expressed under the native promoter was detected at the penetration zone. The V592 transformant expressing VdSCP8-GFP under the native promoter was observed after growth on cellophane for 8 d. Bar = 2.5 μm. (D) The ring signals of SPVdSCP8-GFP, SPVdSCP9-GFP and SPVdSCP10-GFP at the penetration zone. V. dahliae transformants expressing SPVdSCP8-GFP, SPVdSCP9-GFP and SPVdSCP10-GFP under the control of the oliC promotor were used for the assay. The plasma membrane was stained with FM4-64 (red). Bar = 2.5 μm. (PDF) [file ppat.1006275.s004.pdf]

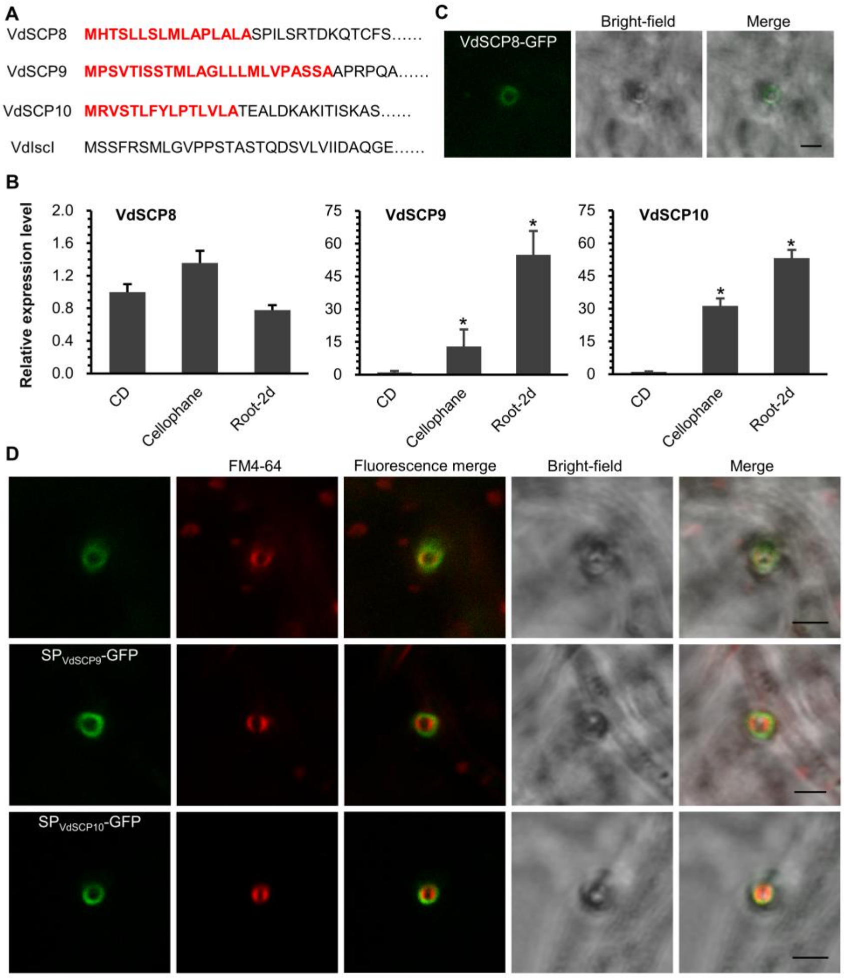

S4 Fig. Secretion of signal peptide containing proteins on the penetration interface. (A) Signal peptide analysis of VdSCP8, VdSCP9, VdSCP10 and VdIscI in *V. dahliae*. The signal peptide of each protein was predicted using the SignalP 4.1 server. The predicted signal peptides are marked in red color, and the 30 amino acids from the initiation codon are displayed. (B) Detection of the expression levels of selected SCPs by qRT-PCR. RNA samples isolated from 2-day-old fungal culture harvested in liquid Czapek-Dox (CD) medium, 4-day-old fungi cultured on cellophane and 2-day-old fungi on cotton roots. The relative expression levels were estimated using the $2^{-\Delta\Delta Ct}$ method. The expression level of each SCP gene in liquid culture was arbitrarily set to 1. The mean and standard errors were calculated from three independent replicates. The asterisks indicate significant differences (*P<0.05; Dunnett's test). (C) VdSCP8-GFP expressed under the native promoter was detected at the penetration zone. The V592 transformant expressing VdSCP8-GFP under the native promoter was observed after growth on cellophane for 8 d. Bar = 2.5 μm. (D) The ring signals of SP_{VdSCP8}-GFP, SP_{VdSCP9}-GFP and $SP_{VdSCP10}$-GFP at the penetration zone. *V. dahliae* transformants expressing SP_{VdSCP8}-GFP, SP_{VdSCP9}-GFP and $SP_{VdSCP10}$-GFP under the control of the oliC promotor were used for the assay. The plasma membrane was stained with FM4-64 (red). Bar = 2.5 μm.
