# Supplementary material for: Secretory proteins are delivered to the septin-organized penetration interface during root infection by Verticillium dahliae
Source: PLoS Pathog. 2017 Mar 10;13(3):e1006275. doi: 10.1371/journal.ppat.1006275 (PMC5362242; doi:10.1371/journal.ppat.1006275)
Supplement: S5 Fig — (A) Physical maps of the VdSCP10 locus and the homologous recombination construct obtained by fusion of the VdSCP10 5′flack, hygromycin B resistance gene cassette and VdSCP10 3′flack. The probe and relative positions of primers used for PCR are indicated. (B) Southern blot analysis of targeted gene deletion mutants. EcoRI digested genomic DNA from the V592 wild type strain and two putative VdΔscp10 transformants were blotted with the probe indicated in the schematic diagram. (C) PCR amplification of genomic DNA from the complemented transformants using the primer pair in-F and in-R produced a banding pattern consistent with the integration of an intact gene in V592. (D) The colony morphology of the wild-type V592 and VdΔscp10 mutant strains and the corresponding complemented strains on PDA plates after a 2-week incubation. (E, F) Disease symptoms (E) and disease grades (F) of cotton plants infected with wild-type V592, VdΔscp10 mutant and the complementary strains at 21 dpi. The disease grade (DG) was calculated as previously described. Four is the highest DG, meaning that the entire plant died, while 0 is the lowest DG with no visible wilting. Three replicates of 36 plants were used for each inoculum (*P<0.05; t-test). (PDF) [file ppat.1006275.s005.pdf]

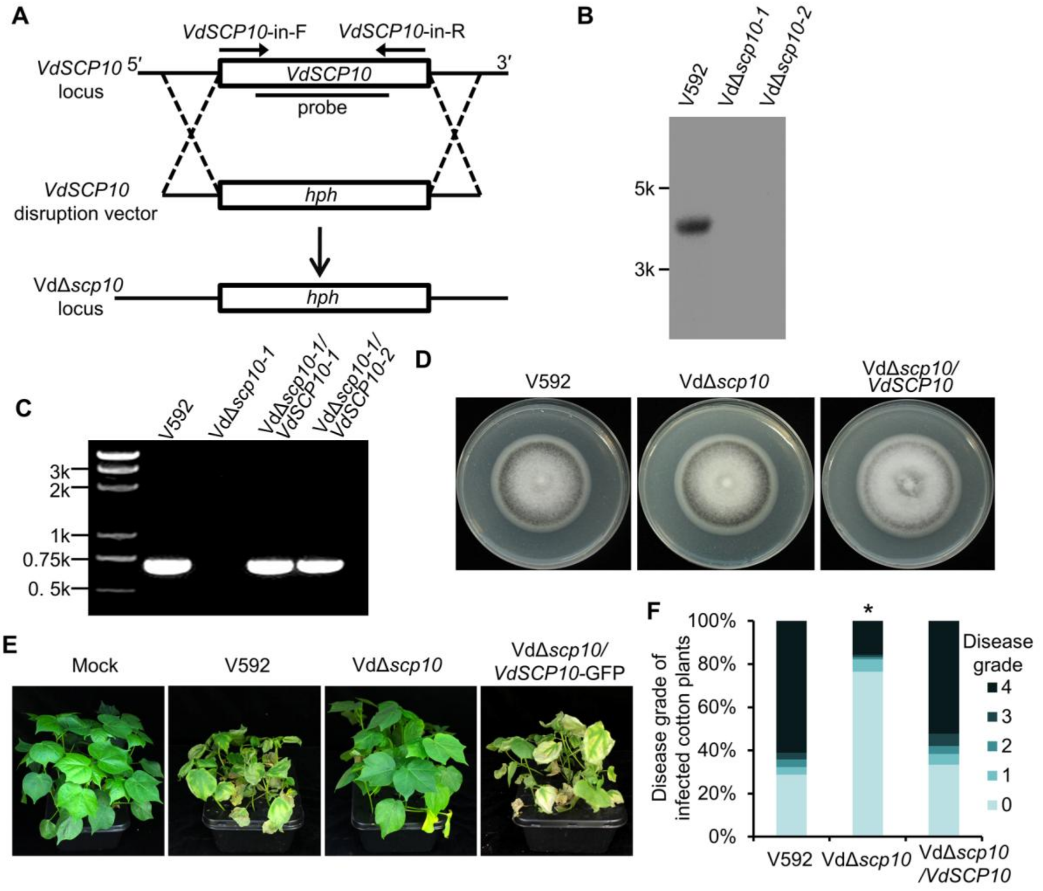

S5 Fig. Targeted deletion of *VdSCP10* decreased virulence of *V. dahliae* in cotton plants. (A) Physical maps of the *VdSCP10* locus and the homologous recombination construct obtained by fusion of the *VdSCP10* 5′flack, hygromycin B resistance gene cassette and *VdSCP10* 3′flack. The probe and relative positions of primers used for PCR are indicated. (B) Southern blot analysis of targeted gene deletion mutants. EcoRI digested genomic DNA from the V592 wild type strain and two putative VdΔ*scp10* transformants were blotted with the probe indicated in the schematic diagram. (C) PCR amplification of genomic DNA from the complemented transformants using the primer pair in-F and in-R produced a banding pattern consistent with the integration of an intact gene in V592. (D) The colony morphology of the wild-type V592 and VdΔ*scp10* mutant strains and the corresponding complemented strains on PDA plates after a 2-week incubation. (E, F) Disease symptoms (E) and disease grades (F) of cotton plants infected with wild-type V592, VdΔ*scp10* mutant and the complementary strains at 21 dpi. The disease grade (DG) was calculated as previously described. Four is the highest DG, meaning that the entire plant died, while 0 is the lowest DG with no visible wilting. Three replicates of 36 plants were used for each inoculum (*P<0.05; t-test).
