# Supplementary material for: Secretory proteins are delivered to the septin-organized penetration interface during root infection by Verticillium dahliae
Source: PLoS Pathog. 2017 Mar 10;13(3):e1006275. doi: 10.1371/journal.ppat.1006275 (PMC5362242; doi:10.1371/journal.ppat.1006275)
Supplement: S6 Fig — (A) Physical maps of the VdSep3 locus and of the homologous recombination constructs obtained by fusion of the VdSep3 5′flack, hygromycin B resistance gene cassette and VdSep3 3′flack. Probes and relative positions of the primers used for PCR are indicated. hph, hygromycin resistance gene. (B) Southern blot analysis of targeted gene deletion mutants. BamHI digested genomic DNA from the V592 strain and two putative VdΔsep3 transformants were blotted with the probe indicated in the schematic diagram. KpnI and HindIII digested genomic DNA from V592 wild type strain and two putative VdΔsep5 transformants were analyzed as described above. (C) PCR amplification of genomic DNA from the complemented transformants using the primer pair in-F and in-R produced a banding pattern consistent with the integration of an intact VdSep3 and VdSep5. Lanes 1–4 were using for the verification of VdΔsep3 complementation and lanes 5–8 were for the verification of VdΔsep5 complementation. (D) Colony morphology of V592, VdΔsep3 and VdΔsep5 mutants and the complementary strains on PDA plates incubated for 2 weeks. (E) Penetration ability analysis of VdΔsep3 and VdΔsep5. Colonies of V592, VdΔsep3 and VdΔsep5 on M0 medium overlaid with cellophane (Before) and removal of the cellophane membrane (After). Images in the first row were obtained at 6 dpi, and the colonies below the cellophane were obtained at 9 dpi. (F) Hyphopodium morphology analysis of V592, VdΔsep3 and VdΔsep5. images were obtained at 6 dpi. (G) Deficient development of the hyphopodium in VdΔsep3 and VdΔsep5. Fungi incubated on cellophane at 5 dpi were used for the observation. The numbers of hyphopodia were counted in three fields of the culture under a light microscope at x1000 magnification with three replicates. The mean and SD for (G) were calculated from three clones for each mutant (*P<0.05; t-test). (H, I) Disease symptoms (H) and disease grades (I) of cotton plants infected with wild-type V592, VdΔsep3 and VdΔsep5 mutants [file ppat.1006275.s006.pdf]

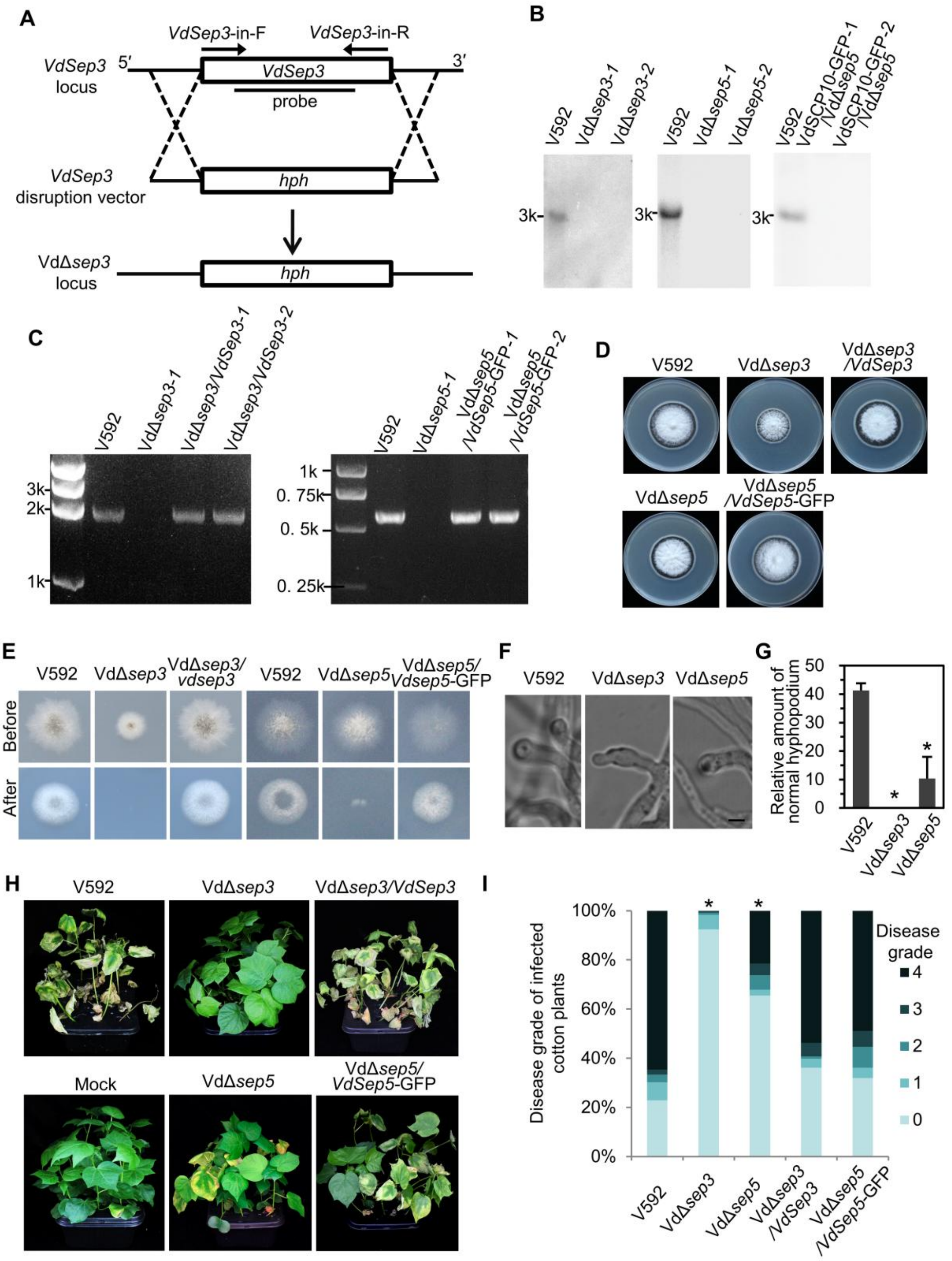

S6 Fig. Targeted deletions of *VdSep3 and VdSep5* genes in *V. dahliae* strain V592. (A) Physical maps of the *VdSep3* locus and of the homologous recombination constructs obtained by fusion of the *VdSep3* 5′flack, hygromycin B resistance gene cassette and *VdSep3* 3′flack. Probes and relative positions of the primers used for PCR are indicated. *hph*, hygromycin resistance gene. (B) Southern blot analysis of targeted gene deletion mutants. BamHI digested genomic DNA from the V592 strain and two putative VdΔ*Sep3* transformants were blotted with the probe indicated in the schematic diagram. KpnI and HindIII digested genomic DNA from V592 wild type strain and two putative VdΔ*Sep5* transformants were analyzed as described above. (C)

PCR amplification of genomic DNA from the complemented transformants using the primer pair in-F and in-R produced a banding pattern consistent with the integration of an intact *VdSep3* and *VdSep5*. Lanes 1-4 were using for the verification of VdΔ*Sep3* complementation and lanes 5-8 were for the verification of VdΔ*Sep5* complementation. (D) Colony morphology of V592, VdΔ*sep3* and VdΔ*Sep5* mutants and the complementary strains on PDA plates incubated for 2 weeks. (E) Penetration ability analysis of VdΔ*Sep3* and VdΔ*Sep5*. Colonies of V592, VdΔ*Sep3* and VdΔ*Sep5* on M0 medium overlaid with cellophane (Before) and removal of the cellophane membrane (After). Images in the first row were obtained at 6 dpi, and the colonies below the cellophane were obtained at 9 dpi. (F) Hyphopodium morphology analysis of V592, VdΔ*Sep3* and VdΔ*Sep5*. Photographs were taken at 6 dpi. (G) Deficient development of the hyphopodium in VdΔ*Sep3* and VdΔ*Sep5*. Fungi incubated on cellophane at 5 dpi were used for the observation. The numbers of hyphopodia were counted in three fields of the culture under a light microscope at x1000 magnification with three replicates. The mean and SD for (G) were calculated from three clones for each mutant (**P*<0.05; t-test). (H, I) Disease symptoms (H) and disease grades (I) of cotton plants infected with wild-type V592, VdΔ*Sep3* and VdΔ*Sep5* mutants and the complementary strains at 21 dpi. Three replicates of 36 plants were used for each inoculum. The asterisks indicate significant differences compared with V592 infection (**P*<0.05; t-test).
