# Supplementary material for: Secretory proteins are delivered to the septin-organized penetration interface during root infection by Verticillium dahliae
Source: PLoS Pathog. 2017 Mar 10;13(3):e1006275. doi: 10.1371/journal.ppat.1006275 (PMC5362242; doi:10.1371/journal.ppat.1006275)
Supplement: S7 Fig — (A) Physical maps of the VdSec22 locus and the homologous recombination construct obtained by fusion of the VdSec22 5′flack, hygromycin B resistance gene cassette and VdSec22 3′flack. The probe and relative positions of primers used for PCR are indicated. The same approach was used for disruption of VdSyn8 and VdExo70. (B) Southern blot analysis of targeted gene deletion mutants. NcoI-digested genomic DNA from V592 and two putative VdΔsec22 transformants were blotted with the probe indicated in the schematic diagram. NcoI-digested genomic DNA from the V592 and two putative VdΔsyn8 transformants were analyzed as described above. SmaI and BamHI-digested genomic DNA from the V592 wild type strain and two putative VdΔexo70 transformants were analyzed as described above. (C) PCR amplification of genomic DNA from the complemented transformants using the primer pair in-F and in-R produced a banding pattern consistent with the integration of an intact gene in V592. Lanes 1–4, 5–8 and 9–12 were for the verification of VdΔsyn8, VdΔsec22 and VdΔexo70 complementation, respectively. (D) Colony morphology of wild-type V592 and VdΔexo70, VdΔsec22 and VdΔsyn8 mutant strains and the corresponding complemented strains on PDA plates 2 weeks post-incubation. (PDF) [file ppat.1006275.s007.pdf]

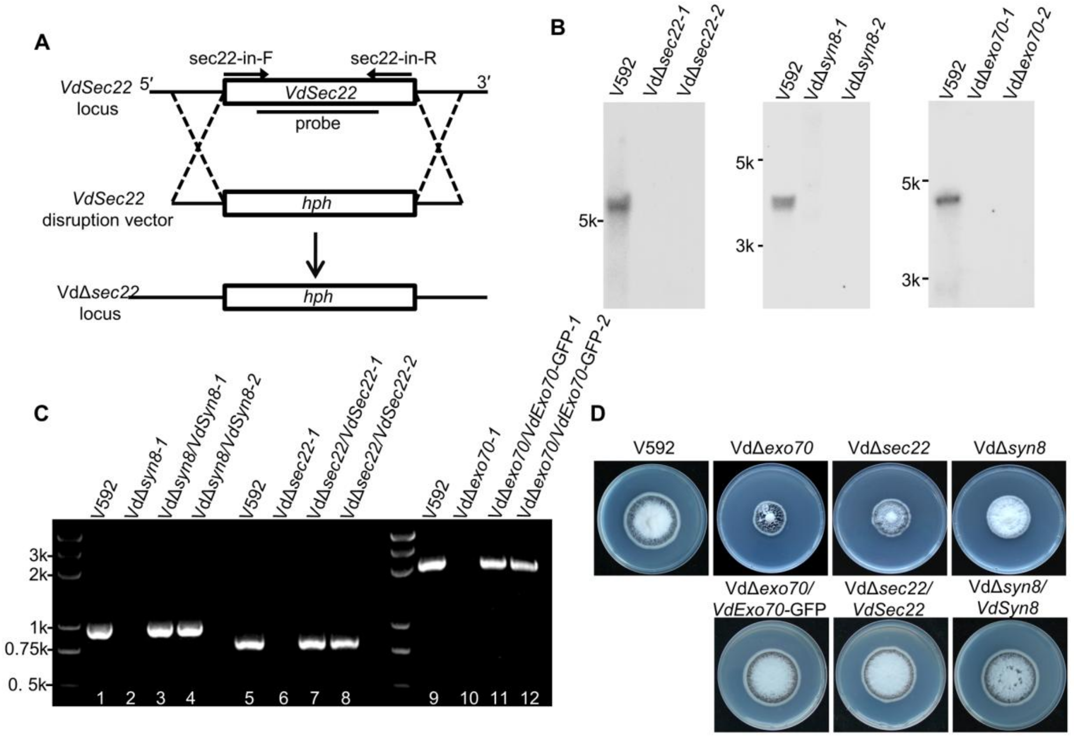

S7 Fig. Gene disruptions of *VdSec22, VdSyn8* and *VdExo70* in *V. dahliae*.
(A) Physical maps of the *VdSec22* locus and the homologous recombination construct obtained by fusion of the *VdSec22* 5′flack, hygromycin B resistance gene cassette and *VdSec22* 3′flack. The probe and relative positions of primers used for PCR are indicated. The same approach was used for disruption of *VdSyn8* and *VdExo70*. (B) Southern blot analysis of targeted gene deletion mutants. NcoI-digested genomic DNA from V592 and two putative VdΔ*sec22* transformants were blotted with the probe indicated in the schematic diagram. NcoI-digested genomic DNA from the V592 and two putative VdΔ*syn8* transformants were analyzed as described above. SmaI and BamHI-digested genomic DNA from the V592 wild type strain and two putative VdΔ*exo70* transformants were analyzed as described above. (C) PCR amplification of genomic DNA from the complemented transformants using the primer pair in-F and in-R produced a banding pattern consistent with the integration of an intact gene in V592. Lanes 1-4, 5-8 and 9-12 were for the verification of VdΔ*syn8*, VdΔ*sec22* and VdΔ*exo70* complementation, respectively. (D) Colony morphology of wild-type V592 and VdΔ*exo70*, VdΔ*sec22* and VdΔ*syn8* mutant strains and the corresponding complemented strains on PDA plates 2 weeks post-incubation.
