# Supplementary material for: Secretory proteins are delivered to the septin-organized penetration interface during root infection by Verticillium dahliae
Source: PLoS Pathog. 2017 Mar 10;13(3):e1006275. doi: 10.1371/journal.ppat.1006275 (PMC5362242; doi:10.1371/journal.ppat.1006275)
Supplement: S8 Fig — Genomic DNA isolated from V592, VdΔsyn8, VdΔsec22 and VdΔexo70 expressing VdSCP10-GFP were digested with EcoRI for Southern blot analysis. Red arrowheads indicate selected colonies with single copy insertions for further study. Hybridization was performed with the 32P-labeled oliC promotor-specific DNA probe as shown below. (PDF) [file ppat.1006275.s008.pdf]

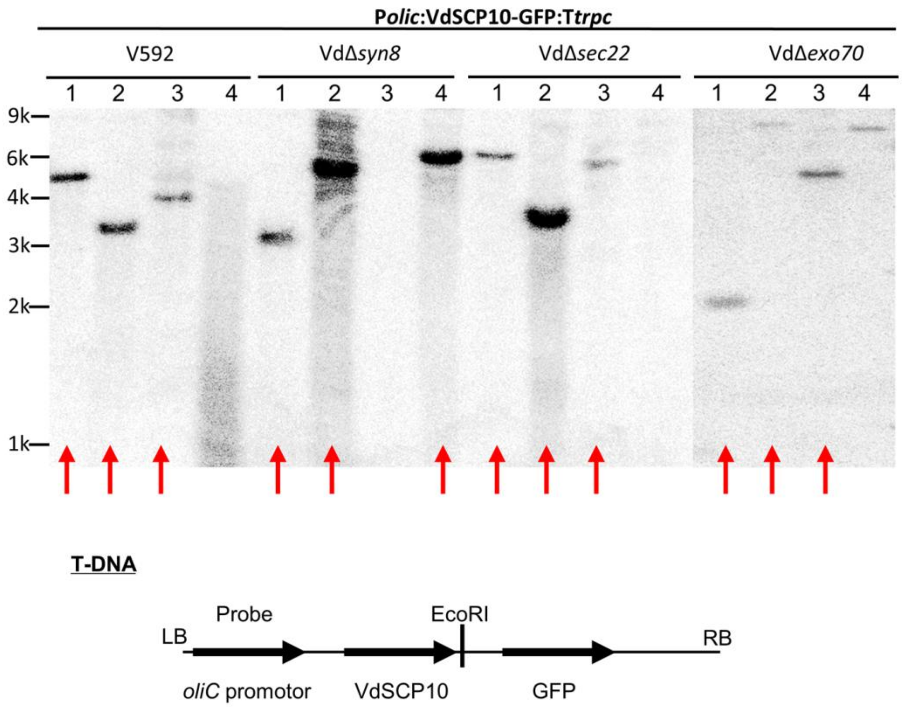

S8 Fig. Determination of the T-DNA insertional copy number of VdSCP10-GFP in V592, VdΔ*syn8*, VdΔ*sec22* and VdΔ*exo70*.

Genomic DNA isolated from V592, VdΔ*syn8*, VdΔ*sec22* and VdΔ*exo70* expressing VdSCP10-GFP were digested with EcoRI for Southern blot analysis. Red arrowheads indicate selected colonies with single copy insertions for further study. Hybridization was performed with the ^{32}P-labeled oliC promotor-specific DNA probe as shown below.
