# Supplementary material for: Secretory proteins are delivered to the septin-organized penetration interface during root infection by Verticillium dahliae
Source: PLoS Pathog. 2017 Mar 10;13(3):e1006275. doi: 10.1371/journal.ppat.1006275 (PMC5362242; doi:10.1371/journal.ppat.1006275)
Supplement: S9 Fig — VdSCP10-GFP expressed under the oliC promotor was transformed into V592 and VdΔsec22. Staining the hyphal ER with ER-Tracker Blue-White DPX. Bar = 2.5μm. (PDF) [file ppat.1006275.s009.pdf]

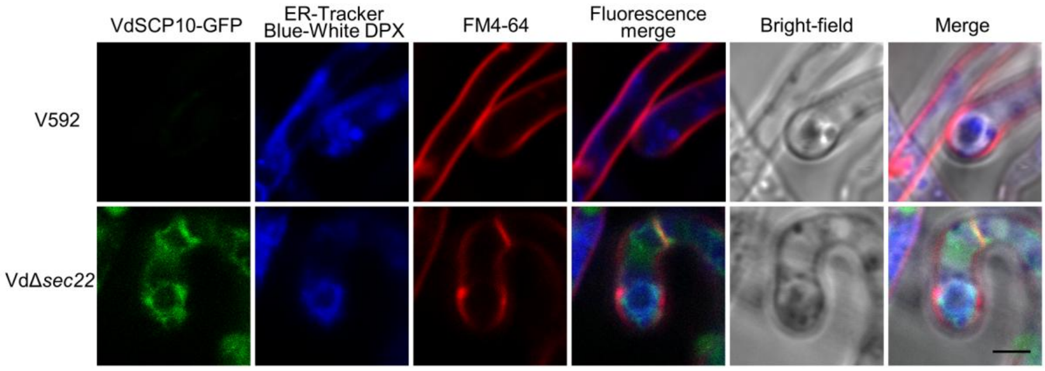

S9 Fig. Deletion of *VdSec22* caused VdSCP10-GFP retention in the ER.
VdSCP10-GFP expressed under the oliC promotor was transformed into V592 and VdΔ*sec22*. Staining the hyphal ER with ER-Tracker Blue-White DPX. Bar = 2.5 μm.
