# Supplementary material for: Secretory proteins are delivered to the septin-organized penetration interface during root infection by Verticillium dahliae
Source: PLoS Pathog. 2017 Mar 10;13(3):e1006275. doi: 10.1371/journal.ppat.1006275 (PMC5362242; doi:10.1371/journal.ppat.1006275)
Supplement: S10 Fig — (A) Localization of VdSec8-GFP and VdExo70-GFP in the hyphal tips of V. dahliae on cellophane. (B) Localization of VdSec8-GFP and VdExo70-GFP at the base of the hyphopodium on cellophane. (C) VdSec8-GFP organized at the base of the hyphopodium on the surface of the root. (D) After invasive hyphae developed, VdSec8-GFP was organized at the hyphal neck on cellophane and plant roots. Bar = 2.5μm. (PDF) [file ppat.1006275.s010.pdf]

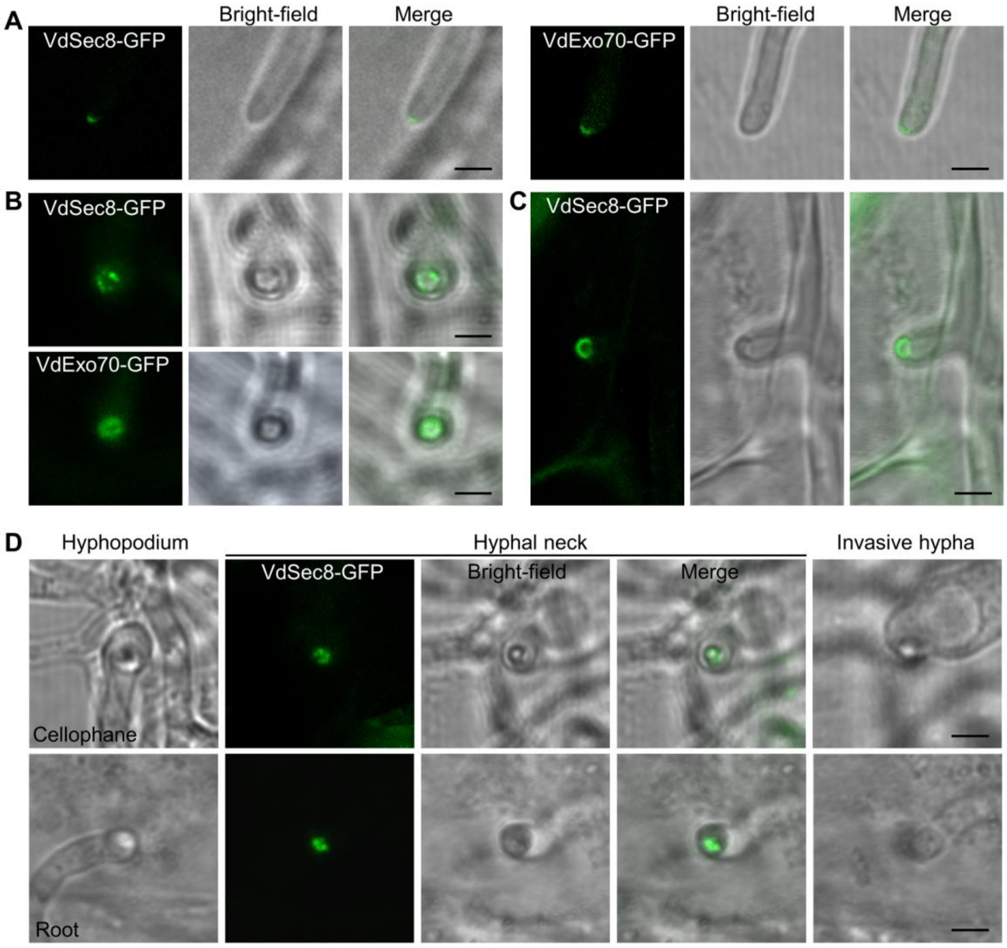

S10 Fig. Localization of *V. dahliae* exocyst subunits at the hyphal tips, hyphopodium base and hyphal neck.

(A) Localization of VdSec8-GFP and VdExo70-GFP in the hyphal tips of *V. dahliae*on cellophane. (B) Localization of VdSec8-GFP and VdExo70-GFP at the base of the hyphopodium on cellophane. (C) VdSec8-GFP organized at the base of the hyphopodium on the surface of the root. (D) After invasive hyphae developed, VdSec8-GFP was organized at the hyphal neckon cellophane and plant roots. Bar = 2.5 μm.
