# Supplementary material for: Secretory proteins are delivered to the septin-organized penetration interface during root infection by Verticillium dahliae
Source: PLoS Pathog. 2017 Mar 10;13(3):e1006275. doi: 10.1371/journal.ppat.1006275 (PMC5362242; doi:10.1371/journal.ppat.1006275)
Supplement: S11 Fig — Confocal laser scanning microscopy (CLSM) images and linescan graph showing co-localization of VdSec8-GFP and VdSep5-RFP at the hyphal neck. Bar = 2.5μm. (PDF) [file ppat.1006275.s011.pdf]

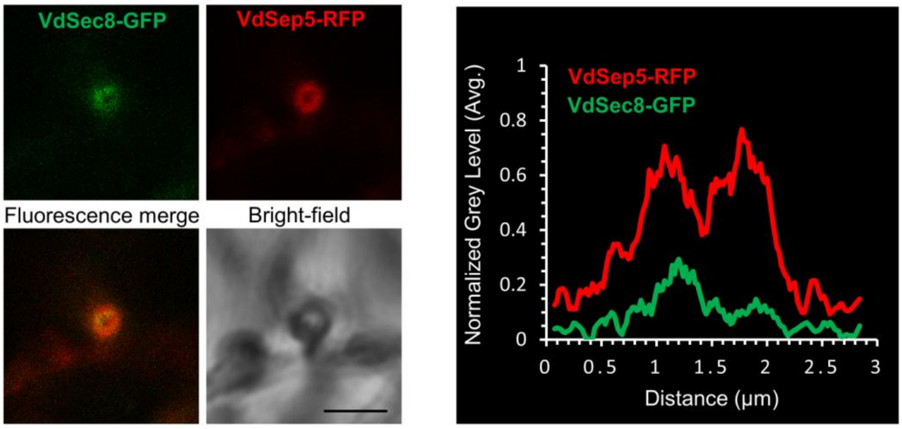

S11 Fig. Colocalization of *V. dahliae* exocyst subunit VdSec8 and VdSep5 at the hyphal neck.

Confocal laser scanning microscopy (CLSM) images and linescan graph showing co-localization of VdSec8-GFP and VdSep5-RFP at the hyphal neck. Bar = 2.5μm.
